# Structural basis of glucosinolate recognition and transport by plant GTR1

**DOI:** 10.1038/s41421-026-00884-7

**Published:** 2026-04-07

**Authors:** Rui Yan, Junping Fan, Cheng Chi, Bowen Zhang, Di Wu, Huiwen Chen, Jianke Gong, Xiaoguang Lei, Daohua Jiang

**Affiliations:** 1 College of Life Science and Technology, Key Laboratory of Molecular Biophysics of MOE, Huazhong University of Science and Technology, Wuhan, Hubei China; 2 Beijing National Laboratory for Condensed Matter Physics and Institute of Physics, Chinese Academy of Sciences, Beijing, China; 3 Beijing National Laboratory for Molecular Sciences, Key Laboratory of Bioorganic Chemistry and Molecular Engineering of Ministry of Education, College of Chemistry and Molecular Engineering, Peking-Tsinghua Center for Life Sciences, New Cornerstone Science Laboratory, Peking University, Beijing, China; 4 Peking University Institute of Advanced Agricultural Sciences, Shandong Laboratory of Advanced Agricultural Sciences at Weifang, Weifang, Shandong China; 5 School of Physical Sciences, University of Chinese Academy of Sciences, Beijing, China

**Keywords:** Cryoelectron microscopy, Plant molecular biology

## Abstract

Glucosinolates (GLSs) play crucial roles in plant defense against herbivores. GTR1 facilitates the high-affinity transport of GLSs through a proton-dependent process. However, the molecular mechanism underlying GLS recognition and transport by GTR1 remains largely unknown. Here, we present four cryo-EM structures of *Arabidopsis* GTR1 in distinct states, namely, the outward-apo, inward-apo, 4MTB-bound and 3IMG-bound forms, revealing the structural basis for GLS and proton cotransport by GTR1. GTR1 consists of an MFS-like transmembrane domain and an intracellular domain (ICD). The ICD plays an essential role in GTR1 function by interacting with the gating helix, transmembrane helix 7. GLSs are recognized by the central cavity residues and directly interact with the conserved E_1_X_1_X_2_E_2_K motif. Our structural and functional analyses demonstrated that the E_1_X_1_X_2_E_2_K motif and Glu513 determine the proton coupling of GTR1. This study provides mechanistic insights into how GTR1 transports GLSs, which could aid in improving crop quality and enhancing resistance to herbivory.

## Introduction

Glucosinolates (GLSs) are a group of secondary metabolites that occur naturally in plants of the order *Brassicales*, including economically important crops such as rapeseed (*Brassica napus*) and mustard (*Brassica juncea*)^1–3^. GLSs share the general chemical structure of a thiohydroximate-O-sulfonate moiety linked to glucose, but contain variable side chains, which are derived from modified aliphatic, aromatic, and indole amino acids, resulting in >200 derivatives identified to date^4–8^. When tissue destruction occurs, myrosinase enzymes break down GLSs to glucose and various metabolites, some of which are toxic and thereby contribute to plant defenses against pathogens and herbivores^9–11^. On the other hand, these GLS derivatives impair nutritional quality and limit the utility of plants as animal feed owing to potential toxic effects or unpleasant flavors^12^. An increasing number of studies suggest that some GLSs have potential cancer-preventive properties^13–15^. The GLS-myrosinase defense system exhibits a compartmentalized distribution. Myrosinase is stored largely in myrosin cells, whereas GLSs are synthesized in maternal tissues and transported to target tissues such as S-cells and seeds^16–18^. The long-distance transport of GLSs is carried out by transporter proteins. In the model plant *Arabidopsis thaliana*, three GLS transporters, GTR1/2/3 (also known as NPF2.10/11/9), play vital roles in GLS-specific tissue accumulation, and loss-of-function mutations to these transporters display phenotypes with altered GLS distributions and reduced GLS accumulation in seeds^19–23^. Moreover, four GTR homologs engineered in *Brassica juncea* were shown to reduce antinutritional GLS metabolites in oilseeds^24^, highlighting the use of GTRs as targets to increase the nutritional value of crops. Consequently, elucidating the mechanism of GLS transport is essential not only for understanding plant defense strategies but also for developing transport engineering approaches for eliminating antinutritional metabolites in high-value crops.

GTRs function as high-affinity proton-coupled symporters^19^ that are responsible for the cellular uptake of GLSs from the apoplast^20^. GTR1/NPF2.10 has been suggested to be a multifunctional transporter that may be involved in nitrate, jasmonic acid-isoleucine (JA-Ile), and gibberellic acid (GA) transport^25^. GTRs belong to the nitrate and peptide transporter family (NPF), which has 53 members in *Arabidopsis*^2,19,26,27^. NPF transporters are critical for plant growth and development because they transport a wide range of substrates across the cell membrane, including nitrate, chloride, potassium, GLSs, and phytohormones^19,28,29^. The NPF in plants and the cognate proton-dependent oligopeptide transporter (POT/PTR) family in bacteria and animals share a highly conserved E_1_X_1_X_2_E_2_R/K motif in transmembrane helix 1 (TM1) that is involved in proton coupling and active transport^26,30–34^. Structural studies of the plant nitrate transporter NRT1.1 (also known as NPF6.3) and peptide transporters revealed that these transporters adopt the common fold of major facilitator superfamily (MFS) transporters and the canonical alternating access transport mechanism^35–40^. However, the underlying mechanism through which GTRs recognize and transport GLSs remains largely unknown. In plants, NRT1.1 is a transceptor that can switch from a low-affinity state to a high-affinity state depending on the phosphorylation status of Thr101, which is conserved in GTRs^39–41^. Conversely, mutating this Thr to Asp, which mimics phosphorylation, abolished the transport activities of GLSs, suggesting a distinct regulatory mechanism^42,43^. The inward-facing structures of NRT1.1 suggested that His356 and Glu476 engage with the E_1_X_1_X_2_E_2_R/K motif, acting as key determinants for proton-coupled transport^39,40^. However, His356 is not conserved in GTRs, and the lack of an NPF structure in the outward-facing state hinders the validation of this hypothesis. Therefore, the proton-coupled transport mechanism of NPF transporters remains incompletely understood. Moreover, NPF members exhibit a large intracellular domain (ICD) between TM6 and TM7 that is not resolved in the NRT1.1 structure, and the functional role of this domain and how it interacts with the TM core are still unknown.

In this study, we determined the cryo-electron microscopy (cryo-EM) structures of *Arabidopsis* GTR1 in distinct functional states, including inward-facing, outward-facing, and GLS-bound states, and elucidated key structural features and conformational changes that occur during state transitions. Combined with mutagenesis and transport analyses, the results of this study revealed the structural basis for GLS recognition and transport by GTR1. We further elucidated that E513 may act as a second protonation site and work in concert with the E_1_X_1_X_2_E_2_K/R motif to govern proton-coupled active transport. Taken together, the results of our study provided mechanistic insights into understanding substrate recognition, proton coupling, and transport by plant NPF transporters.

## Results

### Function and structural determination of GTR1

**Fig. 1:**
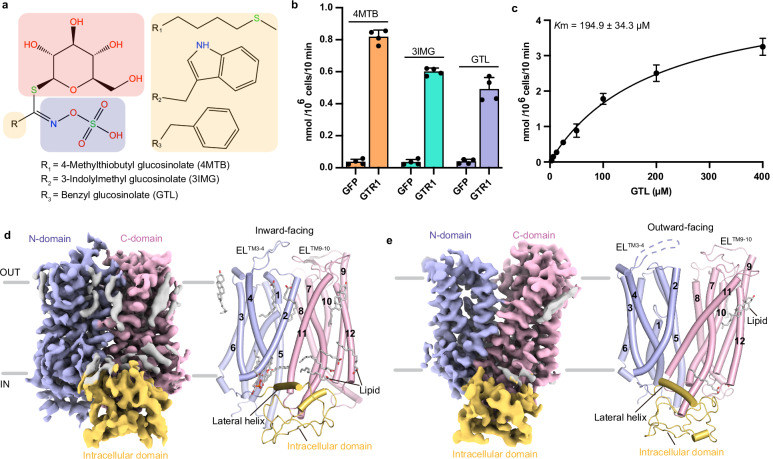
Functional characterization and cryo-EM structures of GTR1. **a** Chemical structures of three representative GLSs used in this study. The variable side chains (R), sugars, and sulfate moieties are shown in orange, red, and purple boxes, respectively. **b** GTR1 transport activity for different substrates. Cells expressing GFP served as controls. The data are presented as the mean ± SEM from *n* = 4 independent replicates. **c** Concentration-dependent uptake of GTL by HEK293F cells transfected with GTR1. The data are presented as the mean ± SEM; *n* = 3–4 independent replicates. **d**, **e** EM maps and cartoon representations of GTR1 in the inward-facing (**d**) and outward-facing states (**e**). The N-domain, C-domain, ICD, and lipids are colored light blue, light pink, light orange, and gray, respectively.

GLSs can be classified into three types according to their variable side chain structures: aliphatic GLSs are derived from alanine, leucine, isoleucine, methionine or valine; benzenic GLSs are derived from phenylalanine or tyrosine; and indolic GLSs are derived from tryptophan^6,7^. We selected three representative GLSs, 4-methylthiobutyl glucosinolate (4MTB), 3-indolylmethyl glucosinolate (3IMG), and benzyl glucosinolate (GTL), to study how *Arabidopsis* GTR1 transports these substrates (Fig. 1a). The GLS uptake activity of wild-type (WT) GTR1 heterologously expressed in human embryonic kidney (HEK) 293 cells was assessed via a liquid chromatography–tandem mass spectrometry (LC–MS/MS) transport assay, which revealed that GTR1-expressing cells exhibited robust uptake of the three GLSs in the order of 4MTB >3IMG > GTL (Fig. 1b), which is consistent with the findings of previous studies^19,43^. We next investigated the concentration-dependent uptake of GTL by GTR1 at pH 5, which yielded a *K*_m_ value of 194.9 ± 34.3 μM (Fig. 1c). This *K*_m_ value is comparable to that in a previous study in which GTR1 was expressed in cotton cell lines^44^. However, these *K*_m_ values are ~10-fold greater than those reported in another study^43^, presumably because the GLS transport assays of GTR1 in this study were performed using different expression systems, pH values, and substrate uptake durations.

To investigate the structural basis of GTR1 transport, we purified WT GTR1 in lauryl maltose neopentyl glycol (LMNG) micelles for cryo-EM single-particle analysis. The size-exclusion chromatography (SEC) profile suggested that GTR1 existed as a mixture of different oligomeric states in solution (Supplementary Fig. S1a, b). The two-dimensional (2D) class averages clearly revealed the presence of both dimeric and monomeric forms within the samples (Supplementary Fig. S1c, d). However, only the monomeric structures were resolved at high resolution. We determined the structures of the apo form of GTR1 in the inward- and outward-facing states at resolutions of 3.2 Å and 3.5 Å, respectively (Fig. 1d, e; Supplementary Figs. S2, S3 and Table S1). Additionally, we resolved the GTR1 structures in complex with substrates 4MTB (GTR1^4MTB^) and 3IMG (GTR1^3IMG^) at resolutions of 3.2 Å and 3.1 Å, respectively (Supplementary Figs. S4, S5 and Table S1). The density maps facilitated the construction of a GTR1 model, which ranged from Ile60 to Lys608, and the placement of lipids and ligands (Supplementary Figs. S2e, S3e, S4e, S5e).

### Architecture of GTR1

The overall architecture of GTR1 consists of a transmembrane core domain (TMD), extracellular loops (ELs), intracellular loops (ILs), and a large ICD, with both the N- and C-terminal ends situated in the cytoplasm. The TMD is characterized by 12 transmembrane (TM) helices that form an N-domain (TM1–TM6) and a C-domain (TM7–TM12) with twofold pseudosymmetry, reminiscent of a typical MFS fold (Fig. 1d, e; Supplementary Fig. S1e). The N- and C-domains form a central cavity where 4MTB and 3IMG bind. The relatively long ELs of TM3–4 (EL^TM3–4^) and TM9–10 (EL^TM9–10^) were clearly resolved in the inward-facing structure, and both adopted a hairpin-like turn and point close to each other (Fig. 1d). However, the density map for the region between Pro161 and Gly172 of EL^TM9–10^ is completely unresolved in the outward-facing structure (Fig. 1e). These observations suggest that these two ELs may contribute to stabilizing the inward-facing state.

**Fig. 2:**
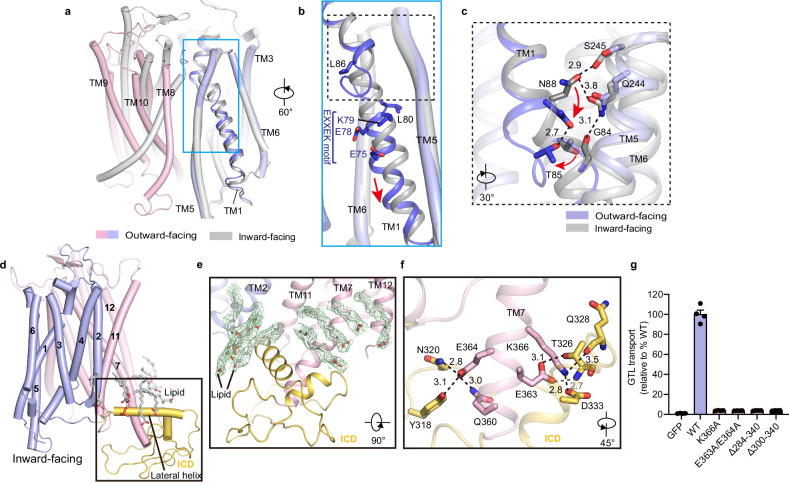
Architecture of GTR1. **a** Superposition of outward-facing and inward-facing states using the N-domain as a reference. The blue box indicates TM1. **b** Conformational shift in TM1 between the outward-facing and inward-facing states. **c** Magnified view of the interactions between TM1 and TM5 in the outward-facing and inward-facing states. The black dashed lines represent polar interactions, and the red arrows indicate the rotation of T85 and N88. **d** The inward-facing GTR1 structure is depicted as a cartoon model with lipids and the ICD in gray and light orange, respectively. The ICD is highlighted in a black box. **e** Lipids bind to the ICD. Lipids are depicted as gray sticks. The EM densities in this figure are shown as green meshes and contoured at 7 σ. **f** Magnified view of the interactions between TM7 and the ICD; the black dashed lines represent potential hydrogen bonds or salt bridges. **g** GTL uptake by GTR1^WT^ and the GTR1 mutants with alterations in TM7 and the intracellular interaction network. Cells expressing GFP served as controls. Data were normalized to the total cell number for each variant and are presented relative to GTR1^WT^. The values are presented as the mean ± SEM of *n* = 4 independent replicates.

A structural comparison of the inward- and outward-facing structures of GTR1 revealed that most TMs retain unchanged helical conformations, whereas TM1 exhibits pronounced conformational rearrangement (Fig. 2a). In the inward-facing GTR1 structure, TM1 assumes a continuous helical form, but it breaks into separate helices in the region of Leu80–Leu86 in the outward-facing structure (Fig. 2b). This structural rearrangement disrupts the interactions between Asn88, Ser245, and Gln244 from TM5, resulting in a downward movement of TM1 (Fig. 2b, c). Importantly, this hinge region in TM1 is located above the conserved E_1_X_1_X_2_E_2_K/R motif (Fig. 2b), suggesting that the hinge in TM1 might play a role in E_1_X_1_X_2_E_2_K/R motif-mediated state transitions.

Structural superposition revealed that the inward-facing GTR1 structure closely resembles the crystal structure of NRT1.1 with a root mean square deviation (r.m.s.d.) of 1.73 Å over 423 aligned Cα atoms (Supplementary Fig. S6a). However, only the amphipathic lateral helix (Pro251–Glu269) of the ICD was modeled in NRT1.1^39,40^, whereas the entire ICD (Pro283–Thr358) was well resolved in both the inward-facing and outward-facing GTR1 structures, allowing a detailed analysis of the interactions between the ICD and the TMD core (Fig. 1d, e; Supplementary Figs. S2e, S3e, S6b). Plant NPF transporters possess a conserved ICD between TM6 and TM7, but the specific function of this ICD remains unclear. Our GTR1 structures revealed that the ICD is situated laterally inferior to the TMD core, forming contacts mainly with TM7 (Fig. 2d). The ICD is organized into three layers: the lateral helix forming the top layer, a pair of hairpin-like antiparallel loops forming the bottom layer, and an additional short helix and the N-terminus of TM7 that fill the space between the two layers (Fig. 2d). We observed several lipid-like densities lying on the top surface of the ICD, which is partly embedded in the lipid membrane (Fig. 2e). The cytosolic extended TM7 forms extensive polar and nonpolar interactions with the ICD. Specifically, Gln360 and Glu364 of TM7 engage in hydrogen bonding interactions with Tyr318 and Asn320 of the ICD and Glu363 and Lys366 of TM7 participate in electrostatic interactions with Asp333 and the backbone carbonyl oxygen of Thr326 of the ICD (Fig. 2f). To validate the functional role of ICD, we generated two ICD-truncated constructs, ΔLeu284–Pro340 and ΔLeu300–Pro340, and two proteins with site-directed mutations, K366A and E363A/E364A, and evaluated their effects on transport activity. As illustrated in Fig. 2g, these four alterations led to completely abolished GTL uptake activity, suggesting that the ICD plays an essential role in GTR1 function. Notably, the protein expression of E363A/E364A, K366A, and the two ICD-truncated mutants was markedly lower than that of WT GTR1 (Supplementary Fig. S8a), suggesting that disrupting the interactions between the ICD and TM7 by performing these mutations may also reduce protein stability.

We next compared our GTR1 structures with those of its bacterial and mammalian homologs. Structural superpositions revealed that the TMD of GTR1 is more similar to those of human PepT1 and PepT2^45^ and horse PepT1^34^ than to those of bacterial homologs^46–48^ (Supplementary Fig. S7a–f). Additionally, substantial local conformational differences were observed. For example, the ICDs between TM6 and TM7 of human PepT1 and PepT2 are much smaller than that of GTR1, whereas this region in bacterial homologs forms two additional transmembrane helices, HA and HB (Supplementary Fig. S7a–h). Moreover, mammalian homologs have a large extracellular domain (ECD), which is suggested to be important for substrate transport^34^; such an ECD is absent in GTR1 and its bacterial homologs (Supplementary Fig. S7a–f). Furthermore, in the outward-facing state, TM1 became two short helices in GTR1, whereas it adopted a continuous helical structure in HsPepT1 (Supplementary Fig. S7i).

### Glucosinolate recognition by GTR1

**Fig. 3:**
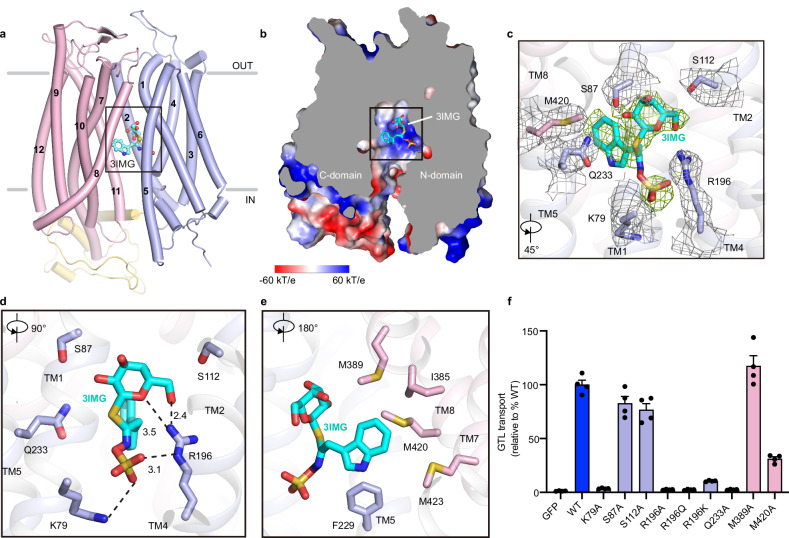
3IMG recognition by GTR1. **a** Structure of 3IMG bound to GTR1 in the inward-facing state. 3IMG is shown in ball and stick representation. The N-domain and C-domain are colored light blue and light pink, respectively. **b** Cut-open electrostatic potential surface of GTR1^3IMG^, with 3IMG shown as sticks. The electrostatic potential surface was calculated using the APBS plug-in in PyMOL (red to blue, −60 kT/e to +60 kT/e). **c** EM density maps of 3IMG and interacting residues of GTR1. The densities of these residues and 3IMG are contoured at 4 σ and are shown as gray and green meshes, respectively. **d**, **e** Detailed interactions between GTR1 and the main backbone (**d**) and side chain (**e**) of 3IMG. The interacting residues of GTR1 and 3IMG are depicted as sticks. **f** GTL uptake by GTR1^WT^ and its variants with mutations in the substrate binding site. GFP-expressing cells served as controls. Data were normalized to the total cell number for each variant and are presented relative to GTR1^WT^. The values are presented as the mean ± SEM of *n* = 4 independent replicates.

GTR1 is responsible for GLS accumulation in seeds and GLS translocation from the apoplast to the phloem for long-distance transport^19,20^. To understand how GTR1 recognizes GLSs, we determined the cryo-EM structures of GTR1 in complex with 4MTB (GTR1^4MTB^) and 3IMG (GTR1^3IMG^), both of which were captured in an inward-facing state (Fig. 3; Supplementary Fig. S9a). GTR1 possesses a positively charged central cavity, where additional strong nonproteinaceous EM densities were observed in GTR1^4MTB^ and GTR1^3IMG^ but not in the apo structures, to accommodate 4MTB and 3IMG (Fig. 3b, c; Supplementary Fig. S9b). The overall structures of GTR1^4MTB^ and GTR1^3IMG^ are essentially identical to that of apo GTR1 in the inward-facing conformation, except for local rotation of some substrate-interacting residues.

The 3IMG-bound and 4MTB-bound structures revealed the precise GLS binding site in GTR1. The glucose and sulfonic acid moieties of 3IMG are positioned in the N-domain, and the indole group is situated mainly in the C-domain (Fig. 3b). Specifically, the negative sulfonic acid group engages in electrostatic interactions with the positive Lys79 on TM1 and Arg196 on TM4; the glucose moiety participates in polar interactions with Ser87, Ser112, Arg196, and Gln233; and the indole group forms hydrophobic and van der Waals interactions with Phe229 from the N-domain and Ile385, Met389, Met420, and Met423 from the C-domain (Fig. 3d, e). Notably, K79 of the conserved E_1_X_1_X_2_E_2_K motif, which is essential for proton coupling and the active transport of nitrate^39,40^, glucosinolates^30^, and peptides^49^, is directly involved in GLS binding. The K79A mutation completely abolished GTL accumulation (Fig. 3f), which is in line with the findings of a previous study^43^. Additionally, substituting charged residue Arg196 with alanine or glutamine completely abolished GTR uptake, and the R196K mutant retained weak transport activity (only ~10% compared with WT GTR1) (Fig. 3f), suggesting that the guanidine head of Arg196, which interacts with both the sugar and sulfonic acid moieties, plays an indispensable role in GLS recognition. The Q233A mutation also fully diminished GTL transport activity, whereas the S87A and S112A mutations caused a minor reduction in activity of less than 20% (Fig. 3f). Moreover, the M389A mutation had no effect on GTL uptake, and M420A reduced GTL uptake by ~70% (Fig. 3f), suggesting that the variable side chains of GLSs are less critical for binding.

Previous studies have suggested that GTR1 has low-affinity nitrate transport activity^19^. Structural alignment of GTR1 with NRT1.1 revealed that although Lys79, Arg196 and E513 are conserved, other key nitrate-coordinating residues, such as His356, Thr360, Tyr388 and Phe511, of NRT1.1 are replaced by Ile385, Met389, Leu419 and Ser548 in GTR1, respectively (Supplementary Fig. S6c), which at least in part contribute to the reduced nitrate transport activity of GTR1 compared with NRT1.1. Additionally, compared with that of NRT1.1, the TM1 of GTR1 is notably displaced by ~4 Å away from the central cavity, which enlarges the central pocket to accommodate bulky GLS substrates (Supplementary Fig. S6d, e). Previous studies have suggested that GTR1 may also transport JA- and GA-hormones^25,43^, and we speculate that the enlarged, partly positive and partly hydrophobic central cavity of GTR1 could serve as a receptor site to which other hormones bind, such as JA-Ile and GA (Supplementary Fig. S6f–h).

Superposition of the ligand binding sites of GTR1^4MTB^ and GTR1^3IMG^ revealed that 4MTB adopts a binding mode very similar to that of 3IMG (Supplementary Fig. S9c). In agreement with this observation, the K79A and M420A mutations caused very similar decreases in the uptake of 4MTB, 3IMG, and GTL (Supplementary Fig. S9d). Previous studies have shown that GTR1 and GTR2 exhibit broad specificity for a wide range of GLSs, whereas GTR3 preferentially transports tryptophan-derived GLSs^19,50^. Our GLS transport results confirmed the substrate specificity of GTR1–3 (Supplementary Fig. S9e). Sequence alignment and structural superposition of GTR1^4MTB^ with the AlphaFold2 model of GTR3 demonstrated that the key residues Lys79, Ser87, Arg196, and Gln233, which interact with the consensus GLS backbone, are strictly conserved in GTR1–3; however, the side chain interacting residues are highly conserved in GTR1 and GTR2 but not in GTR3 (Supplementary Figs. S9f, g, S11). For example, the residues Ile385, Met389, Phe540 and Ala544 of GTR1 are Tyr343, Thr347, Tyr498 and Ile502, respectively, in GTR3 (Supplementary Fig. S9g). To investigate which residues are important for 3IMG specificity in GTR3, we generated the four-site GTR1 mutant I385Y/M389T/F540Y/A544I and the reciprocally swapped GTR3 mutant Y343I/T347M/Y498F/I502A. Unexpectedly, the tetrad mutant of GTR1 exhibited broad specificity for all three GLSs, similar to WT GTR1, whereas the tetrad mutation in GTR3 completely abolished GLS uptake (Supplementary Fig. S9h). A recent study systemically investigated 12 nonconserved residues in the central cavity of GTR3 relative to those in GTR1, showing that substituting 9 or 10 of the 12 residues (including Y343I and Y498F) with those from GTR1 abolished the 3IMG specificity of GTR3^51^, suggesting that the substrate specificity of GTRs is determined by multiple residues within the central cavity.

### Structural basis of GTR1 proton coupling

**Fig. 4:**
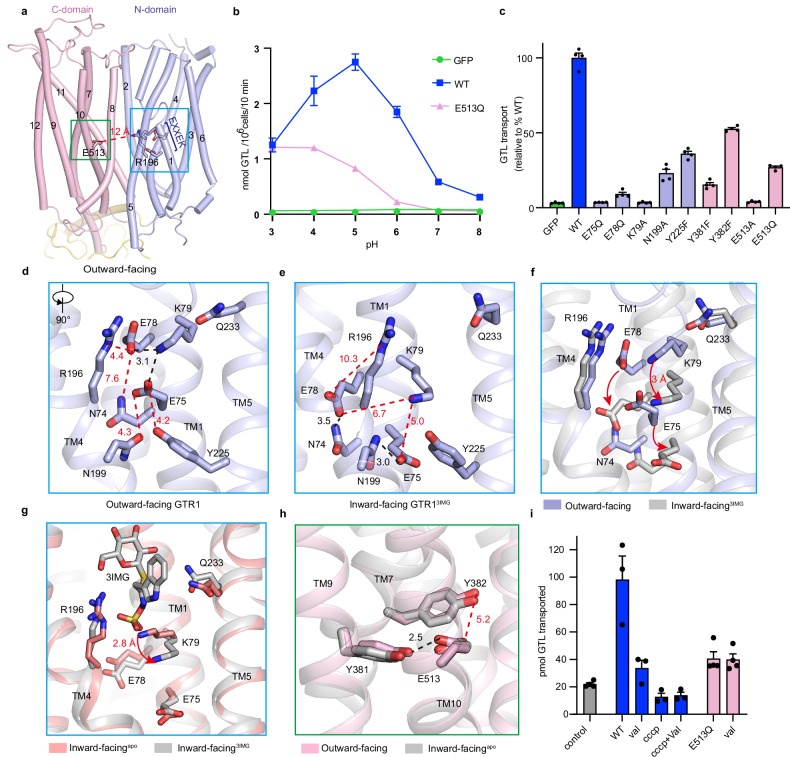
Structural basis for proton coupling in GTR1. **a** Structure of GTR1 in the outward-facing state. The residues involved in proton coupling are depicted as sticks on the N-domain (light blue) and C-domain (light pink). The red dashed line represents the distance between R196 and E513. **b** The pH-dependent GTL transport activity of GTR1^WT^. The cells were incubated under the indicated pH conditions in the presence of 100 μM GTL for 10 min. The data are presented as the mean ± SEM; *n* = 4 independent replicates. **c** GTL uptake by GTR1^WT^ and mutants by HEK293F cells transfected with the indicated protein. GFP served as a control. Data were normalized to the total cell number for each variant and are presented relative to GTR1^WT^. The values are presented as the mean ± SEM of *n* = 4 independent replicates. **d**, **e** The interaction network of the E_1_X_1_X_2_E_2_K motif in outward-facing (**d**) and inward-facing (**e**) states. The black dashed lines indicate hydrogen bonding or electrostatic interactions, and the red dashed lines represent the distances. **f** Conformational shift in the E_1_X_1_X_2_E_2_K motif between the outward-facing and inward-facing states. Red arrows indicate the rotation of E75, E78, and K79. **g** Conformational shift in the E_1_X_1_X_2_E_2_K motif between apo- and 3IMG-bound structures in the inward-facing state. GTR1^apo^ and GTR1^3IMG^ are shown in light red and gray, respectively. The red arrow indicates the rotation of K79. **h** Details of the interactions between E513 and Y381 and Y382 in the outward-facing and inward-facing states. **i** GTL uptake by proteoliposomes containing WT GTR1 or the E513Q variant. The data are presented as the mean ± SEM; *n* = 3–4 independent replicates. val, valinomycin.

GTR1 is known as a proton–GLS symporter^26,27,30^ that occurs via an electrogenic process that is mediated partly by the conserved E_1_X_1_X_2_E_2_K motif. Studies of POTs have suggested a proton-coupling model in which the E_1_X_1_X_2_E_2_K motif may interact with the conserved Glu476 in TM10 in the outward-facing conformation, and His356 in TM7 of plant NRT1.1 and His61 in TM2 of bacterial PepT_So_ may also be involved in proton coupling^52,53^. However, our GTR1 structures revealed that Lys79 of the E_1_X_1_X_2_E_2_K motif and Glu513 (equivalent to Glu476 in NRT1.1) are separated by ~12 Å and ~14 Å in the outward-facing and inward-facing states, respectively, which are too far apart to establish meaningful interactions (Fig. 4a). Additionally, His356 of NRT1.1 is not conserved in GTR1 and is replaced by the hydrophobic residue Ile385. Therefore, the proton-coupled transport mechanism of GTR1 remains incompletely understood.

To investigate the proton-coupling mechanism in GTR1, we first measured GTL uptake by GTR1 at six different pH values (ranging from 3.0 to 8.0). Consistent with previous findings^19,43^, our pH-responsive transport assay revealed that GTR1 exhibited optimal transport activity at pH 5.0 and that the activity decreased sharply when the pH exceeded this value (Fig. 4b). The E75Q, E78Q, and K79A mutations in the E_1_X_1_X_2_E_2_K motif in addition to the E513A mutation almost completely eliminated GTL uptake, whereas E513Q retained ~25% of the transport activity of WT GTR1 at pH 5.0 (Fig. 4c). Unlike that of WT GTR1, the pH-responsive transport results revealed that the optimum pH of E513Q was lower than that of the WT, with maximal uptake activity occurring at pH 3.0–4.0 (Fig. 4b). These functional results indicate that both the E_1_X_1_X_2_E_2_K motif and Glu513 are involved in the pH dependency of GTR1.

We next analyzed the detailed interactions and local changes in the conformations of the E_1_X_1_X_2_E_2_K motif and Glu513 in GTR1 structures captured in different states (Fig. 4d–h). Lys79 interacts with both Glu75 and Glu78 in the outward-facing GTR1 structure, but Glu75 and Glu78 shift away from Lys79 in the inward-facing state; instead, Glu75 participates in hydrogen bonding interactions with Asn199 and Tyr255, and Glu78 interacts with Asn74 (Fig. 4d, e). Superposition of the N-domains of the two structures revealed that TM1 in the inward-facing GTR1 structure shifts downward ~3 Å relative to that in the outward-facing structure because of the helix breakage (Figs. 2f, 4f). In addition, the inward-facing conformations of the apo- and 3IMG-bound structures are highly similar, with the notable exception of the displacement of Lys79 (Fig. 4g). Consequently, residues in the E_1_X_1_X_2_E_2_K motif undergo conformational shifts during state transitions and reestablish interactions with surrounding residues (Fig. 4d–g). The N199A and Y255F mutations strongly impaired GTL uptake by approximately 75% and 60%, respectively (Fig. 4c). On the other hand, Glu513 forms hydrogen bonding interactions with Tyr381 on TM7, an ion pair that has been found in proton-coupled MFS transporters such as human vesicular monoamine transporter 2 (VMAT2)^54,55^. Tyr382 is also positioned close to Glu513 (Fig. 4h). The Y381F and Y382F mutations, which eliminate the polar interactions with Glu513, largely reduced GTL uptake by approximately 85% and 50%, respectively (Fig. 4c), suggesting that Tyr381 is more critical for interaction with Glu513 than Tyr382 is. Notably, the Glu‒Tyr interaction is conserved in GTR1–3 but not in NRT1.1, in which the conserved Glu476 forms an ion pair with His356 and plays an essential role in proton-coupled nitrate uptake^39,40^.

To further understand the role of Glu513 in proton coupling, we performed a proteoliposome transport assay. We purified protein samples of the WT GTR1 and E513Q variants and reconstituted the purified samples, as indicated by SDS-PAGE and fluorescence-detection SEC (fSEC) analyses, into proteoliposomes (Supplementary Fig. S8c–e). The proteoliposome transport assays revealed that WT GTR1 exhibited substantially greater GTL uptake than the E513Q mutant did (Fig. 4i). The activity of WT GTR1 was greatly decreased by the addition of the protonophore CCCP or the potassium ionophore valinomycin, indicating that GTR1 is driven by both the proton chemical gradient (ΔH^+^) component and the membrane potential (ΔΨ) component of the proton electrochemical gradient (ΔμH^+^); however, valinomycin had no effect on E513Q, suggesting that the remaining activity of E513Q is driven by the proton chemical gradient (ΔH^+^) rather than the membrane potential (ΔΨ) (Fig. 4i).

In light of the results of previous studies^54,56^, we further performed molecular dynamics (MD) simulations to investigate the effects of protonation on protein dynamics. The outward- and inward-facing GTR1 structures were reconstituted into a lipid bilayer and served as the initial models for MD analysis. Because Glu75/Glu78 of the E_1_X_1_X_2_E_2_K motif and Glu513 are potential protonation sites, MD simulations with targeted protonation of key glutamate residues (Glu78 alone, Glu78/Glu75, and Glu78/Glu75/Glu513) were performed to elucidate the proton-coupled transport mechanism. For both conformations, the overall structures did not experience large domain–domain movements across three independent MD simulations (each 1 μs in duration) (Supplementary Fig. S10a, b). However, we found that protonation affects the interactions of key residues, which may serve as a trigger for state transitions in a physiological context. In the unprotonated outward-facing state, Glu78/Glu75 engage in salt bridge interactions with Lys79 at distances of 2–3 Å; in contrast, protonation of either Glu75 or Glu78 significantly increased their distance to Lys79 (Glu75–Lys79 and Glu78–Lys79), thereby disrupting these salt bridge interactions of the E_1_X_1_X_2_E_2_K motif (Supplementary Fig. S10c). This releases Lys79, priming it for substrate binding, which is consistent with our substrate-bound structures. Additionally, protonation of Glu513 disrupted its hydrogen bonding interactions with Tyr381 and Tyr382 (Supplementary Fig. S10d), which may have contributed to the shift in the conformational equilibrium toward the inward-facing state.

On the basis of these results, we speculated that the E_1_X_1_X_2_E_2_K motif and Glu513 play different roles in proton-coupled GLS transport by GTR1. Protonation of Glu75 and/or Glu78 potentially disrupts the salt bridge networks of the E_1_X_1_X_2_E_2_K motif, which not only induces conformational changes in TM1 but also liberates Lys79 and Arg196, which can then participate in GLS recognition by forming electrostatic interactions with the negative sulfonic acid moiety (Fig. 4d–g). In line with this hypothesis, a previous MD simulation study suggested that protonation of Glu56 of the E_1_X_1_X_2_E_2_K motif in PepT2 facilitates substrate binding^56^. In contrast, Glu513 does not directly interact with substrates in the structures of GTR1 or in NRT1.1^39,40^. Instead, protonation of Glu513 may catalyze the transition of GTR1 from the outward-facing ground state to the inward-facing excited state, which is energized by the membrane potential^38,57^. Subsequently, deprotonation of the E_1_X_1_X_2_E_2_K motif and Glu513 occurs in the inward-facing conformation, thereby facilitating substrate release and a conformational switch back to the outward-facing ground state. The permanently protonated E513Q cannot be deprotonated in the inward-facing state, which may change the transport kinetics of the mutant, as evidenced by the altered pH dependency and insensitivity to valinomycin treatment (Fig. 4b, i).

### Transport mechanism of GTR1

**Fig. 5:**
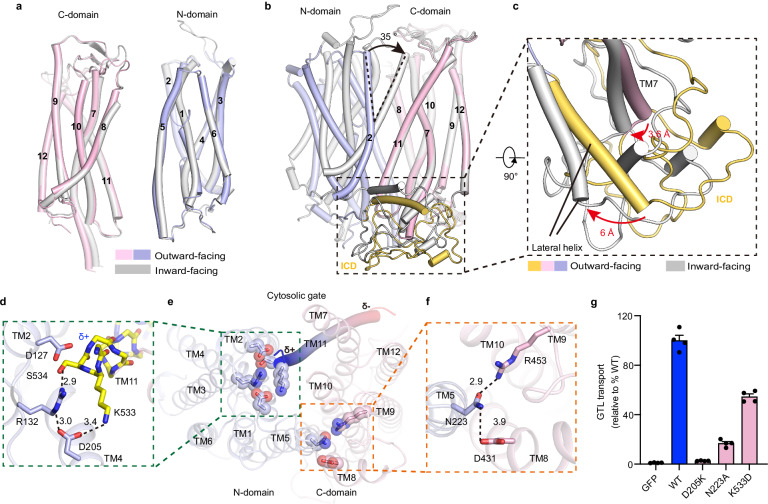
Structural basis of GTR1 state transitions. **a** Structural comparisons of the N- and C-domains of the inward- and outward-facing GTR1 structures. **b** Superposition of outward- and inward-facing states using the C-domains as a reference. The black arrows indicate the oscillations of the C-domains. The black box indicates the ICD. **c** Conformational shifts in TM7 and the ICD between the outward- and inward-facing states. Red arrows indicate the conformational shift of TM7 and the lateral helix. **d**–**f** Interaction network in the intracellular gate of the outward-facing state. The green (**d**, **e**) and orange (**f**) dashed lines outline the areas of interest in the outward-facing state panels. The black dashed lines indicate hydrogen bonding or electrostatic interactions. **g** GTL uptake by GTR1^WT^ and its mutants with altered intracellular interaction networks. Cells expressing GFP served as controls. Data were normalized to the total cell number for each variant and are presented relative to GTR1^WT^. The values are presented as the mean ± SEM of *n* = 4 independent replicates.

To gain insight into the transport dynamics of GTR1, we compared the N- and C-domains of GTR1 in different conformational states. The N-domain or C-domain of the outward-facing GTR1 can be well superimposed with those of the inward-facing structure (Fig. 5a). When the two structures were aligned with the C-domain as a reference, we observed that the N-domain oscillated by up to 35° (Fig. 5b). Notably, TM7 shifted ~3.6 Å between the two structures, and consequently, the lateral helix of the ICD experienced a conformational displacement of ~6 Å (Fig. 5c). These structural observations suggested that GTR1 adopts a canonical alternating access transport mechanism^58,59^.

In the outward-facing state, the intracellular gate is fully closed by TM4–5 of the N-domain and TM10–11 of the C-domain. In particular, we observed several specific interactions that stabilized the outward-facing conformation (Fig. 5d–f). For example, TM11 packs tightly against TM2 on the cytosolic side, where Asp127^TM2^ forms a charge‒dipole interaction with the N-terminus of TM11, and an electrostatic interaction network is formed by Asp127, Arg132, Asp205, Lys533, and Ser534 (Fig. 5d). Additionally, Asn223 engages in polar interactions with both Asp431 and Arg453 (Fig. 5f). Notably, these residues are conserved among GTRs (Supplementary Fig. S11). To validate the functional roles of these interactions, we evaluated GTL uptake by the D205K, K533D, and N223A mutants. The charge-reversed D205K mutation almost completely eliminated GTR1 transport activity, whereas the N223A and K533D variants retained only ~20% and ~50% activity, respectively, compared with that of GTR1^WT^ (Fig. 5g). These results revealed that these specific interactions play important roles in maintaining conformational equilibrium and that mutations disrupting these interactions lead to a deficiency in GTR1 function.

**Fig. 6:**
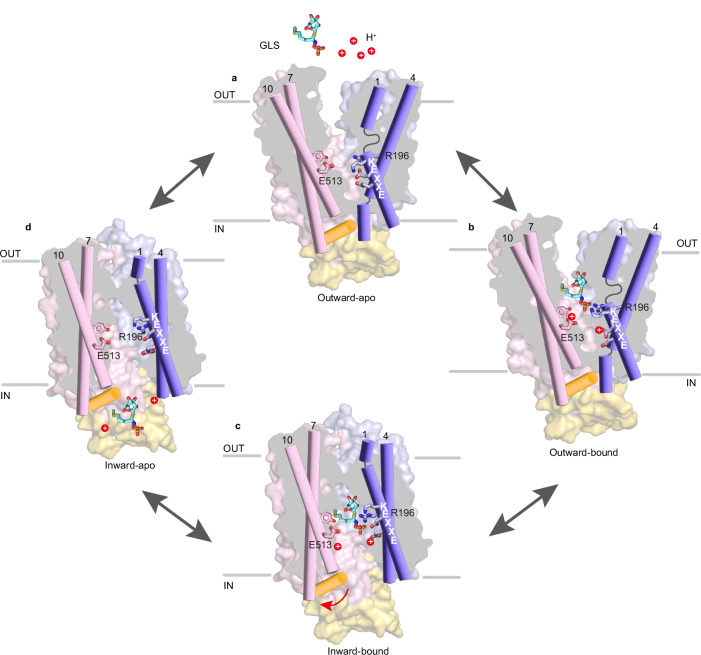
Schematic representation of the GTR1 transport cycle. **a** In the outward-facing conformation, the transporter exposes a large positively charged central cavity accessible to extracellular GLSs and protons. **b** Protonation of Glu75/Glu78 (in the E_1_X_1_X_2_E_2_K motif) enables GLS binding and leads to an occluded state. Subsequent protonation of Glu513 shifts the equilibrium toward the inward-facing conformation. **c** In the transition to the inward-facing state, protons dissociate from the protein, which facilitates GLS release from the transporter. **d** The unloaded transporter resets to the outward-facing state.

Overall, we propose a working model for proton–GLS cotransport by GTR1 (Fig. 6). Transport starts with an outward-facing conformation, where the transporter exposes its positively charged central cavity toward the acidic apoplast (Fig. 6a). GLS and protons access the central cavity, and protonation of Glu75 and/or Glu78 of the E_1_X_1_X_2_E_2_K motif induces local conformational changes in TM1, which not only enable substrate binding but also elicit a state transition to a potentially occluded state. Additionally, protonation of Glu513 further shifts the conformational equilibrium toward an inward-facing state (Fig. 6b). After adopting the inward-facing state, the central cavity opens to the weakly basic cytosol and protons rapidly dissociate from the protein, which may also facilitate the release of GLS from the transporter (Fig. 6c). Consequently, unloaded GTR1 switches back to the outward-facing ground state (Fig. 6d).

## Discussion

Glucosinolates are important secondary metabolites that are involved in plant defense and the edibility of economically important crops^1–3^. Glucosinolates are actively transported to targeted tissues by GTR1–3 and are therefore potential engineering targets for improving crop quality or enhancing plant defense^19,20^. In this study, we elucidated the architecture and transport mechanism of *Arabidopsis* GTR1. Our cryo-EM structures in distinct functional states, combined with site-directed mutagenesis and functional assays, revealed mechanistic insights into GLS recognition and transport by GTR1. The TM core domain of GTR1 closely resembles that of NRT1.1, adopting the classic MFS fold that enables the translocation of diverse substrates across the membrane via an alternating access mechanism^39,40^. However, how the conserved ICD of plant NPF members interacts with the TM core domain and regulates transport remains elusive^39,40^. For the first time, we revealed the structure and function of the ICD between TM6 and TM7, demonstrating that the membrane-anchored ICD participates in extensive interactions with the cavity lining TM7 (Fig. 2d–f). Removal of the ICD and mutation of key residues inactivate GTR1, highlighting that the ICD acts as a scaffold to maintain protein stability and conformational integrity. Notably, this ICD is not conserved in bacterial or mammalian POT members^34,45,46,49,60,61^, suggesting that the ICD-TM7 interface plays a unique role in regulating the transport cycle of plant NPF transporters, a mechanism distinct from that of bacterial or mammalian homologs.

Our 4MTB-bound and 3IMG-bound GTR1 structures revealed a conserved substrate binding pocket in its central cavity. The glucose and sulfonic acid moieties present in all GLSs are recognized by highly conserved residues exclusively from the N-domain, including Lys79 of the E_1_X_1_X_2_E_2_K motif and Arg196 of TM4. Similar interactions have been described for peptide recognition but not nitrate binding^39,40^. Additionally, the variable side chains of GLSs form hydrophobic interactions with multiple residues, mainly from the C-domain, some of which are not conserved in GTR1–3. However, the results from this study and a previous study^51^ suggest that the preference of GTR3 for transporting tryptophan-derived GLSs is likely determined by a combination of variable residues in the central cavity. The structural superposition of GTR1 and NRT1.1 revealed that several key residues in the nitrate binding site are not conserved, explaining their distinct substrate specificities (Supplementary Figs. S6, S11).

The signature E_1_X_1_X_2_E_2_K/R motif of the POT family has long been known to be involved in proton coupling^26,30^. Our GTR1 structures in different conformations revealed that this motif undergoes local conformational rearrangement, potentially facilitating substrate binding and state transitions. We proposed that the E_1_X_1_X_2_E_2_K motif and the conserved Glu513 in TM10 act as two synergistic protonation sites that determine the proton coupling of GTR1: protonation of Glu75 and/or Glu78 of the E_1_X_1_X_2_E_2_K motif disrupts salt bridge interactions, freeing Lys79 to bind GLSs and triggering conformational changes in TM1 that are essential for state transitions; additionally, Glu513 forms an ion pair with Tyr381, and protonation of this site drives electrogenic transport, as E513Q retains ΔpH dependence but loses sensitivity to changes in the membrane potential (ΔΨ). Interestingly, Tyr381 is not conserved in NRT1.1, and residue Glu476 that is present instead possibly interacts with a nonconserved His356, which is involved in proton coupling^39,40^. Notably, our GTR1 structures revealed that Glu513 does not directly interact with Lys79 of the E_1_X_1_X_2_E_2_K motif in either the outward-facing or the inward-facing conformation.

Previous studies revealed that NRT1.1 can switch from a low-affinity transporter to a high-affinity transporter upon phosphorylation of Thr101, either via a dimerization mechanism or a local structural disruption mechanism^39–41^. This threonine residue is conserved in GTR1-3 (Thr135 in GTR1). In contrast to NRT1.1, the T135D mutation almost completely ablated GTL uptake, whereas the activity of the T135A mutation was similar to that of WT GTR1 in HEK293F cells (Supplementary Fig. S8f). fSEC analysis revealed that compared with the T135A and GTR1^WT^ proteins, T135D exhibited a higher oligomeric state (Supplementary Fig. S8g). Our results are in line with previous findings showing that the T135D mutation abolished GLS uptake and altered the membrane localization of the transporter^42,43^.

In summary, this work provides mechanistic insights into GLS transport in plants, elucidating the structural basis for the functional roles of the ICD, substrate recognition, and proton-coupled transport. These insights position GTR1 as a promising engineering target for optimizing the spatial distribution of GLS metabolites — a critical step toward breeding crops with increased resilience and nutritional quality.

## Materials and methods

### Glucosinolate uptake assay

All mutations in GTR1 were introduced by standard site-directed mutagenesis methods using the WT construct as a template. P2 viruses were collected as follows. HEK293F cells cultured in OPM-293 medium (OPM) were infected with 1% (v/v) P2 viruses of WT GTR1 or its variants when the cell density reached 2.5 × 10^6^ cells per ml in a 37 °C incubator with 5% CO_2_ and rotation at 120 rpm. P2 virus expressing GFP alone was used as a negative control to evaluate the effect of the virus on the cells. After 12 h, sodium butyrate (final concentration of 10 mM) was added to the culture to promote protein expression. Cells were harvested 30 h post infection for functional assays. Prior to harvesting, the density of cells expressing GTR1 (or the GFP control) was measured. The cells were then collected by centrifugation at 100× *g* for 2 min and washed once with a prewarmed acidic buffer (90 mM NaCl, 1 mM KCl, 1 mM MgCl_2_, 1 mM CaCl_2_, and 5 mM MES)^30,62^. Cells were resuspended in the same buffer supplemented with 100 μM substrate (GTL, 4MTB or 3IMG). The GTL uptake experiment was carried out at 37 °C for 10 min and terminated by centrifugation of the cells at 100× *g* for 2 min. The cell pellet was subsequently washed three times with ice-cold PBS (pH 8.0) to completely remove the extracellular substrate. The washed cell pellet was lysed by adding 40 μL of 2% (v/v) Triton X-100 and incubating at room temperature for 10 min. Subsequently, 160 μL of ice-cold 80% methanol was added. The mixture was vortexed thoroughly and incubated at room temperature for 30 min to precipitate proteins and extract metabolites. The extract was centrifuged at 15,000× *g* for 30 min at 4 °C to remove debris. A 100 μL aliquot of the supernatant was subjected to LC-MS/MS analysis. Quantification was achieved using analyte-specific calibration curves (concentration range: 0.39–25 μM). The internal standard 4MTB (1.5 μM) was added to both the standards and the samples. Uptake values for all GTR1 variants were first normalized to the total cell number of each sample and then expressed as a percentage of the GTR1WT control. Data are presented as the mean ± SEM from *n* = 4 independent biological replicates.

### Reconstitution into liposomes and transport assays

GTR1 was functionally reconstituted into liposomes composed of a 3:1 palmitoylole-oylphosphatidylethanolamine:1-palmitoyl-2-oleoyl-sn-glycero-3-phospho-(1′-rac-glycerol) (POPE:POPG) (MCE) mixture with Bio-Beads SM-2 following previously described procedures^63,64^. The lipid mixture was dried under reduced pressure using a rotary evaporator to form a homogeneous thin film, ensuring the complete removal of residual chloroform. The film was then hydrated in reconstitution buffer (20 mM HEPES, 150 mM KCl, pH 7.4) to a final lipid concentration of 20 mg/mL by repeated cycles of freezing in liquid nitrogen and thawing at 37 °C. The resulting multilamellar vesicles were extruded 37 times through a 0.4 μm filter to form large unilamellar vesicles (LUVs). The preformed liposomes were treated with 10 mM n-dodecyl-β-D-maltoside (DDM; Anatrace, USA) for 1 h at room temperature. Purified GTR1 (or the E513Q mutant) was reconstituted at a protein:lipid ratio of 1:50 and incubated on ice for 1 h to allow incorporation. The detergent was removed by sequential addition of three batches of Bio-Beads. After slowly agitating at 4 °C for 2 h, a second batch of Bio-Beads was added and incubated for another 2 h. To completely remove the detergent, a third batch of Bio-Beads was added and incubated overnight at 4 °C. After the Bio-Beads were removed, the proteoliposomes were harvested by centrifugation at 120,000× *g* for 30 min at 4 °C. The pellet was resuspended in reconstitution buffer to a final protein concentration of ~1 mg/mL. Control liposomes (no protein) were prepared in parallel by adding an equivalent volume of protein-free SEC buffer during the incorporation step.

For transport measurements, proteoliposomes (or control liposomes) were diluted in the desired external buffer (20 mM MES, pH 5.5 or HEPES, pH 7.4, each containing 150 mM KCl) supplemented with 100 μM GTL. Where indicated, the potassium ionophore valinomycin (1 μM) or the protonophore CCCP (1 μM) was included. Valinomycin was used to dissipate the membrane potential (ΔΨ), thereby allowing validation of the role of the proton gradient (ΔpH) on GTR1 activity. The samples were incubated at room temperature for 15 min and then pelleted by centrifugation at 120,000× *g* for 30 min at 4 °C. The pellet was subsequently washed twice with cold buffer (20 mM MES and 150 mM KCl, pH 5.5) to ensure complete removal of the external substrate. The harvested liposomes were lysed by the addition of methanol. After being incubated at room temperature for 10 min, the samples were centrifuged at 15,000× *g* for 10 min to remove lipid debris. An aliquot (100 μL) of the supernatant was collected for LC-MS/MS quantification. Calibration curves for absolute quantification were prepared daily using pure analyte standards. The standard curve of each type of GLS was established by using 0.023, 0.047, 0.094, 0.188, 0.375, 0.75, 1.5, and 3 μM samples. The internal standard 4MTB (1 μM) was added to both the standards and the samples.

### Quantification by LC-MS/MS

LC-MS/MS was employed for the transport assay. Analyses were performed on a Shimadzu HPLC system equipped with LC-20AD pumps and a SIL-20AC autosampler. Separation was achieved on an ACQUITY UPLC^®^ HSS T3 column (2.1 × 100 mm, 1.8 μm; Waters) using a binary mobile phase consisting of water with 0.1% formic acid (A) and methanol with 0.1% formic acid (B) at a flow rate of 0.2 mL/min. A 6 min gradient was applied as follows: 0–1 min, 5% B; 1–4 min, 95% B; and 4–6 min, 5% B. The injection volume was 1 μL. Mass spectrometric detection was carried out on an AB Sciex Triple Quad 5500 system equipped with an electrospray ionization (ESI) source. The quantification of GTL, 3IMG, and 4MTB was performed in multiple reaction monitoring (MRM) mode by analyzing the 408/97, 447/97, and 420/97 m/z peaks, respectively^65^.

### Protein cloning, expression and purification

Full-length *Arabidopsis* GTR1 was subcloned and inserted into a pEG2 vector, followed by an HRV 3 C protease site, a green fluorescent protein (GFP) and a Twin-Strep tag using the ClonExpress Ultra One Step Cloning Kit V3 (Vazyme Biotech Co., Ltd.). GTR1 was expressed in HEK293F cells using a Bac-to-Bac baculovirus expression system (Invitrogen). HEK293F cells were transfected with P2 virus at a ratio of 1:100 (virus:HEK293F, v:v). After 12 h, the cultures were supplemented with 10 mM sodium butyrate (Sigma, USA). The cells were collected after 60 h and stored at −80 °C.

For GTR1 purification, the cell pellets were resuspended and homogenized in buffer A (20 mM HEPES (pH 7.5), 150 mM NaCl and 2 mM β-mercaptoethanol (β-ME)) supplemented with a protease inhibitor cocktail including 1 mM phenylmethylsulfonyl acid acyl fluoride (PMSF), 0.8 μM pepstatin, 2 μM leupeptin and 2 μM aprotinin. The cell lysate was then centrifuged at 158,600× *g* for 30 min to sediment the crude membranes, which were homogenized and solubilized for 90 min at 4 °C in buffer A supplemented with protease inhibitor cocktail, 1% (w/v) n-dodecyl-β-D-maltoside (DDM; Anatrace, USA) and 0.15% (w/v) cholesterol hemisuccinate (CHS; Anatrace, USA). The insoluble material was removed by ultracentrifugation at 158,600× *g* for 30 min, and the supernatant was incubated with Strep-Tactin beads (Smart-Life Sciences, China). The bound GTR1 protein was then washed with buffer A containing 0.01% (w/v) LMNG and 0.001% (w/v) CHS and finally eluted with the same buffer containing biotin. The GFP and Twin-strep tag was cleaved by PreScission protease (1:25 w/w ratio) on ice for 2 h. After tag removal, the samples were concentrated with a 100 kDa cutoff concentrator (Cat# UFC810096; Merck Millipore) and subjected to size-exclusion chromatography using a Superpose 6 10/300 GL column (Cat# 29-0915-96; Cytiva) equilibrated in buffer A supplemented with 0.005% (w/v) LMNG and 0.0005% (w/v) CHS. The peak was collected and concentrated to ~15 mg/mL for cryo-EM data acquisition. For GTR1^4MTB^ and GTR1^3IMG^, 4MTB and 3IMG were added to the protein solution at a concentration of 1 mM and incubated on ice for 30 min before cryo-EM sample preparation.

### Cryo-EM sample preparation and data acquisition

The purified samples were centrifuged at 15,600× *g* (M1324R, RWD) for 20 min at 4 °C before cryo-EM sample preparation. Quantifoil Cu 1.2/1.3 (300 mesh) grids were glow-discharged (15 mA for 60 s) using a PELCO easiGlo instrument (Ted Pella) before being applied to 4 μL of concentrated GTR1 sample. The grids were blotted with filter paper for 3.5–5 s after 10 s in a Vitrobot Mark IV (Thermo Fisher Scientific) at 100% humidity at 4 °C and vitrified in liquid ethane at the temperature of liquid nitrogen. The grids were loaded onto a 300 kV Titan Krios microscope operated at 300 kV with a K3 Summit direct electron detector (Gatan) and a GIF Quantum energy filter for data collection. All movie stacks were automatically acquired using EPU v3 (Thermo Fisher Scientific). A nominal magnification of 105,000× was used for imaging, yielding a pixel size of 0.85 Å on the images. Each micrograph was recorded with a total dose of ∼60 e^–^/Å^2^. The defocus range was set from –1.0 μm to –2.0 μm.

### Cryo-EM data processing

All the datasets were processed with CryoSPARC (v4.4.1)^66^. For GTR1^outward^, a total of 1176 movie stacks were motion corrected using Patch Motion Correction, and the CTF values were estimated using Patch CTF Estimation. Initial particle selection by blob extraction yielded 1,104,702 particles (box size = 256 px), which underwent multiple rounds of 2D classification to select 648,901 particles for two rounds of resolution-gradient hetero-refinement using a 20 Å low-pass-filtered ab initio model as a reference. The selected 2D class averages from the blob-picked particles then served as templates for template particle selection. After 2 rounds of 2D classification, 589,231 selected particles were subjected to another resolution gradient hetero-refinement. Merging the best particles from both hetero-refinements and performing a final hetero-refinement step yielded a total of 122,091 particles from the best model, which were re-extracted with a box size of 320 pixels for subsequent 3D classification. The selected particles from 3D classification underwent nonuniform refinement, and the resulting map was used as a reference for a final hetero-refinement of all 122,091 particles, yielding 54,982 high-quality particles. The final reconstruction was obtained through nonuniform refinement with local refinement using a mask, achieving an overall resolution of 3.54 Å. During initial processing, the inward-facing state was observed at low resolution, and we collected additional data for the inward-facing state. A total of 1983 movie stacks were collected, and the data were processed similarly. After blob-picking and 2D classification, a total of 943,530 particles were subjected to three rounds of hetero-refinement. The best map generated was low-passed to 15 Å and 20 Å and input as a reference for the next round of hetero-refinement. The final reconstruction map was refined to 3.22 Å from a total of 152,376 particles. A detailed flowchart of the data processing workflow is presented in Supplementary Figs. S2 and S3, respectively.

The GTR1^4MTB^ and GTR1^3IMG^ data were processed similarly to the GTR1^outward^ and GTR1^inward^ data. In total, 1379 and 2988 movie stacks were collected for GTR1^4MTB^ and GTR1^3IMG^, respectively. The final maps for GTR1^4MTB^ at 3.20 Å and GTR1^3IMG^ at 3.14 Å were obtained from 103,209 and 152,376 particles, respectively. A detailed flowchart of the data processing workflow is presented in Supplementary Figs. S4 and S5.

### Model building and refinement

The initial GTR1 model was predicted by AlphaFold2, fitted into the cryo-EM density map of GTR1 in Chimera^67^, and manually inspected and adjusted in Coot^68^. The corrected model was further refined in PHENIX^69^. Ligand CIF files were generated in Phenix using eLBOW. After model refinement, the ligands and lipids were fitted to the extra densities within the maps. For GTR1^4MTB^ and GTR1^3IMG^, the GTR1^inward^ model was similarly fitted into their respective density maps in Chimera, followed by manual correction in Coot and refinement in PHENIX using the same protocol. The cryo-EM data collection and refinement are summarized in Supplementary Table S1.

All figures were prepared with ChimeraX^70^ and PyMOL (Schrödinger, LLC).

### MD simulations of GTR1

Specific protonation states were initially assigned to the cryo-EM structure of GTR1 at physiological pH (7.4) with the CHARMM-GUI Membrane Builder^71^, which was subsequently employed to embed the protein into a 3:1 POPC:POPE phospholipid bilayer with a target lateral dimension of 10 nm × 10 nm. With respect to the inward-facing (IF) and outward-facing (OF) conformations of GTR1, the bilayer was consistently composed of 186 POPC and 62 POPE lipid molecules to maintain structural uniformity across different conformational states. The membrane–protein complex was subsequently solvated in an orthorhombic simulation box (approximate dimensions: 9.9 × 9.9 × 10.8 nm) using GROMACS 2024.5^72^, yielding a system with ~23,000 solvent molecules, with minor variations among replicate simulations. To mimic physiological ionic strength, 0.15 M NaCl was incorporated into the solvated system, with counterions added to neutralize the overall charge^73^. The topological parameters for the protein were generated using the AMBER ff14.SB force field^74^, whereas the GAFF2 force field^75^ was employed for ligand parameterization, with partial charges determined via the RESP fitting method^76^. Unbiased MD simulations of the outward state were conducted for 1 μs in triplicate for each of the six experimental conditions, with all three replicate simulations performed in parallel. The simulation parameters were identical to those utilized for the extended Cα-restrained equilibration step, and all the atomic restraints were removed to enable unconstrained conformational dynamics^77^. Collectively, these unbiased MD simulations yielded a cumulative 6 μs of production sampling data for subsequent analysis. Post-simulation analyses were performed utilizing GROMACS built-in tools to characterize protein structural dynamics and intermolecular interactions. The root mean square deviation (RMSD) of the protein Cα atoms relative to the initial equilibrated structure was computed using gmx rms to evaluate overall conformational stability^78^. The root mean square fluctuation (RMSF) of individual amino acid residues was calculated with gmx rmsf to assess residue-specific flexibility, with fluctuations normalized across the entire production trajectory^79^. Furthermore, interresidue distances — including those between key interaction sites and active-site residues — were quantified using the gmx distance, and time-series data were averaged across replicates to minimize random fluctuations^80^.

### Reporting summary

Further information on research design is available in the Nature Research Reporting Summary linked to this article.

## Supplementary information

Supplementary Figures S1–11 and Table S1

reporting summary

## Data Availability

The four-dimensional cryo-EM density maps of *Arabidopsis* GTR1^outward^, GTR1^inward^, GTR1^4MTB^ and GTR1^3IMG^, have been deposited into the Electron Microscopy Data Bank under accession numbers EMD-64181 (https://www.emdataresource.org/EMD-64181), EMD-64185 (https://www.emdataresource.org/EMD-64185), EMD-64193 (https://www.emdataresource.org/EMD-64193) and EMD-64204 (https://www.emdataresource.org/EMD-and EMD-64204), respectively. The coordinates of *Arabidopsis* GTR1^outward^, GTR1^inward^, GTR1^4MTB^ and GTR1^3IMG^ have been deposited into the Protein Data Bank under accession codes 9UI1 (10.2210/pdb9UI1/pdb), 9UI6 (10.2210/pdb9UI6/pdb), 9UIF (10.2210/pdb9UIF/pdb), and 9UIT (10.2210/pdb9UIT/pdb), respectively.
