# Supplementary material for: Structural basis of glucosinolate recognition and transport by plant GTR1
Source: Cell Discov. 2026 Apr 7;12:26. doi: 10.1038/s41421-026-00884-7 (PMC13056971; doi:10.1038/s41421-026-00884-7)
Supplement: Supplementary file 1 — Supplementary Figures S1–11 and Table S1 [file 41421_2026_884_MOESM1_ESM.pdf]

Supplementary information for
Structural basis of glucosinolate recognition and transport in plant GTR1

Rui Yan[1,2#], Junping Fan[3#], Cheng Chi[4#], Bowen Zhang[2,5], Di Wu[2,5], Huiwen Chen[2], Jianke Gong[1*], Xiaoguang Lei[3*], Daohua Jiang[2,5*]

[1] College of Life Science and Technology, Key Laboratory of Molecular Biophysics of MOE, Huazhong University of Science and Technology, Wuhan, Hubei, China

[2] Beijing National Laboratory for Condensed Matter Physics and Institute of Physics, Chinese Academy of Sciences, Beijing 100190, China

[3] Beijing National Laboratory for Molecular Sciences, Key Laboratory of Bioorganic Chemistry and Molecular Engineering of Ministry of Education, College of Chemistry and Molecular Engineering, Peking-Tsinghua Center for Life Sciences, New Cornerstone Science Laboratory, Peking University, Beijing, 100871, China.

[4] Peking University Institute of Advanced Agricultural Sciences, Shandong Laboratory of Advanced Agricultural Sciences at Weifang, Weifang, Shandong 261000, China

[5] School of Physical Sciences, University of Chinese Academy of Sciences, Beijing 100190, China

# These authors contributed equally to this project.

*Correspondence author

Emails: jiangdh@iphy.ac.cn (D.J.); jiankeg@hust.edu.cn (J.G.); xglei@pku.edu.cn (X.L.)

This file includes:

Supplementary Fig. S1-11

Supplementary Table S1

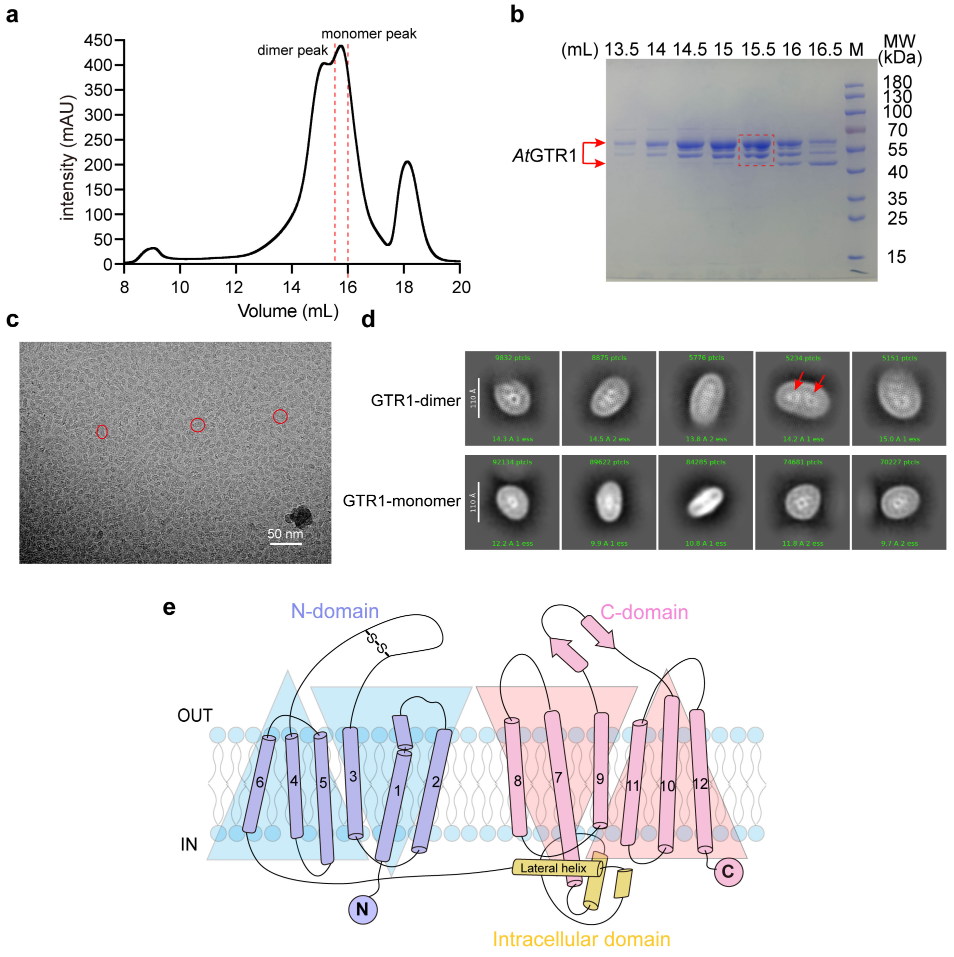

Supplementary Fig. S1. Purification, cryo-EM analysis and topology of Arabidopsis GTR1.

a, Representative size-exclusion chromatography (SEC) profile of the detergent purified GTR1. Peak fractions used for cryo-EM analysis are indicated by red dashed lines. **b,** Peak fractions from SEC were visualized by SDS-PAGE with Coomassie blue staining. Red arrow indicates the bands of GTR1. Red dashed box indicates GTR1 samples used for cryo-EM analysis. The SEC profile and gel image are representative of 3 experimental replicates. **c,** A representative motion-corrected EM micrograph of GTR1. **d,** Reference-free 2D class averages of dimer and monomer. **e,** Topology of GTR1. The intracellular domain (ICD), N- and C-domain are colored in light blue, light pink, and light orange, respectively. The same color scheme is applied throughout the manuscript unless specified.

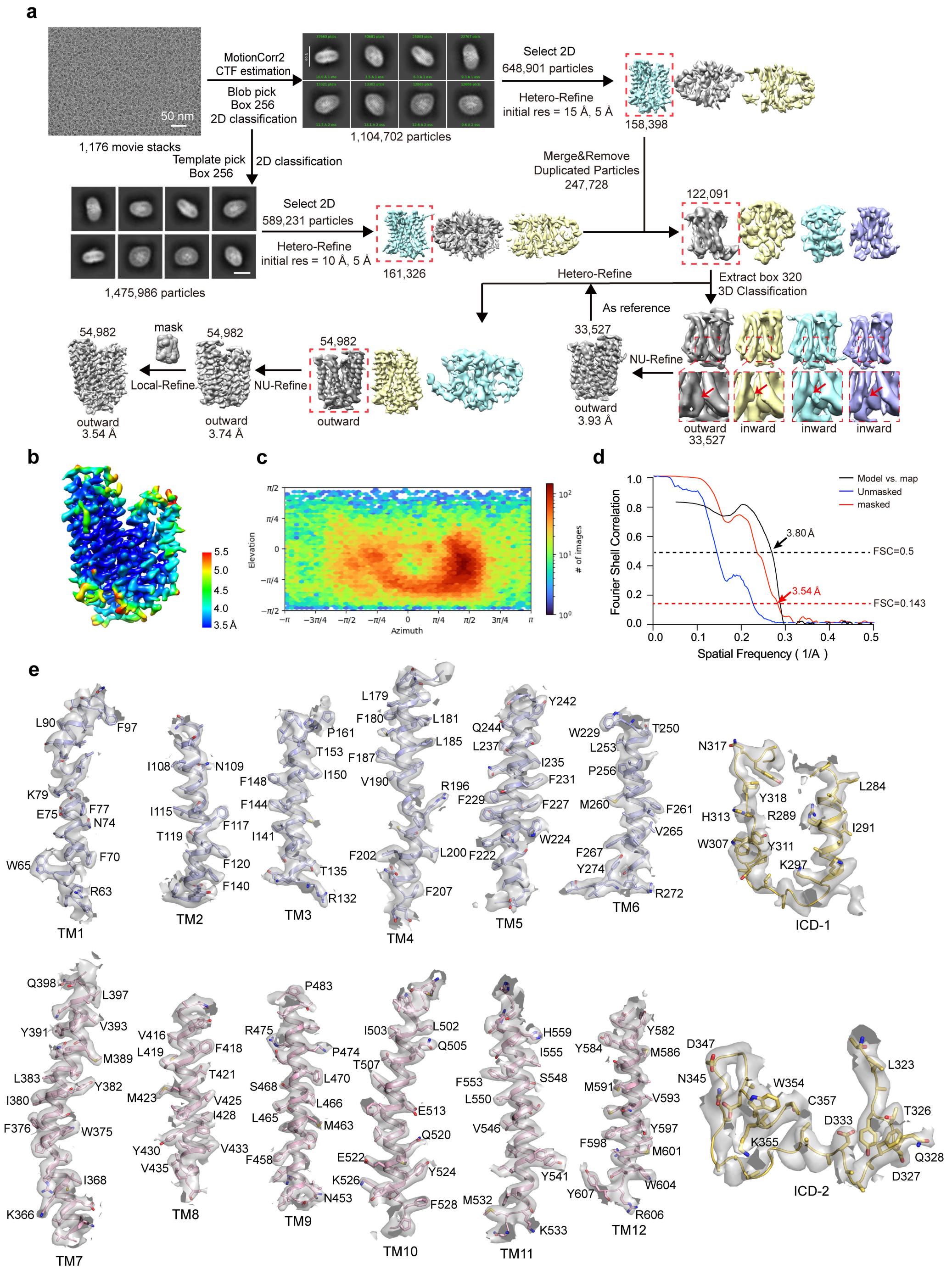

Supplementary Fig. S2. Cryo-EM data processing of GTR1^{outward}.

a, The flowchart for cryo-EM data processing of GTR1^{outward}. A representative motion-corrected EM micrograph of GTR1^{outward} (out of 1,176 micrographs). Scale bar, 50 nm. Scale bar in reference-free 2D class averages is 10 nm. **b, c** and **d,** Local resolution distribution (left panel), particle angular distribution calculated in cryoSPARC for the final reconstruction (middle panel) and FSC curve of the final maps (right panel) of GTR1^{outward}. **e,** Representative EM densities of GTR1^{outward}. EM densities are show in half-transparent surface. Side chains are shown as sticks and labelled. The map contour levels are at 6.5-8 σ with a carving range of 2.2-2.6 Å, respectively.

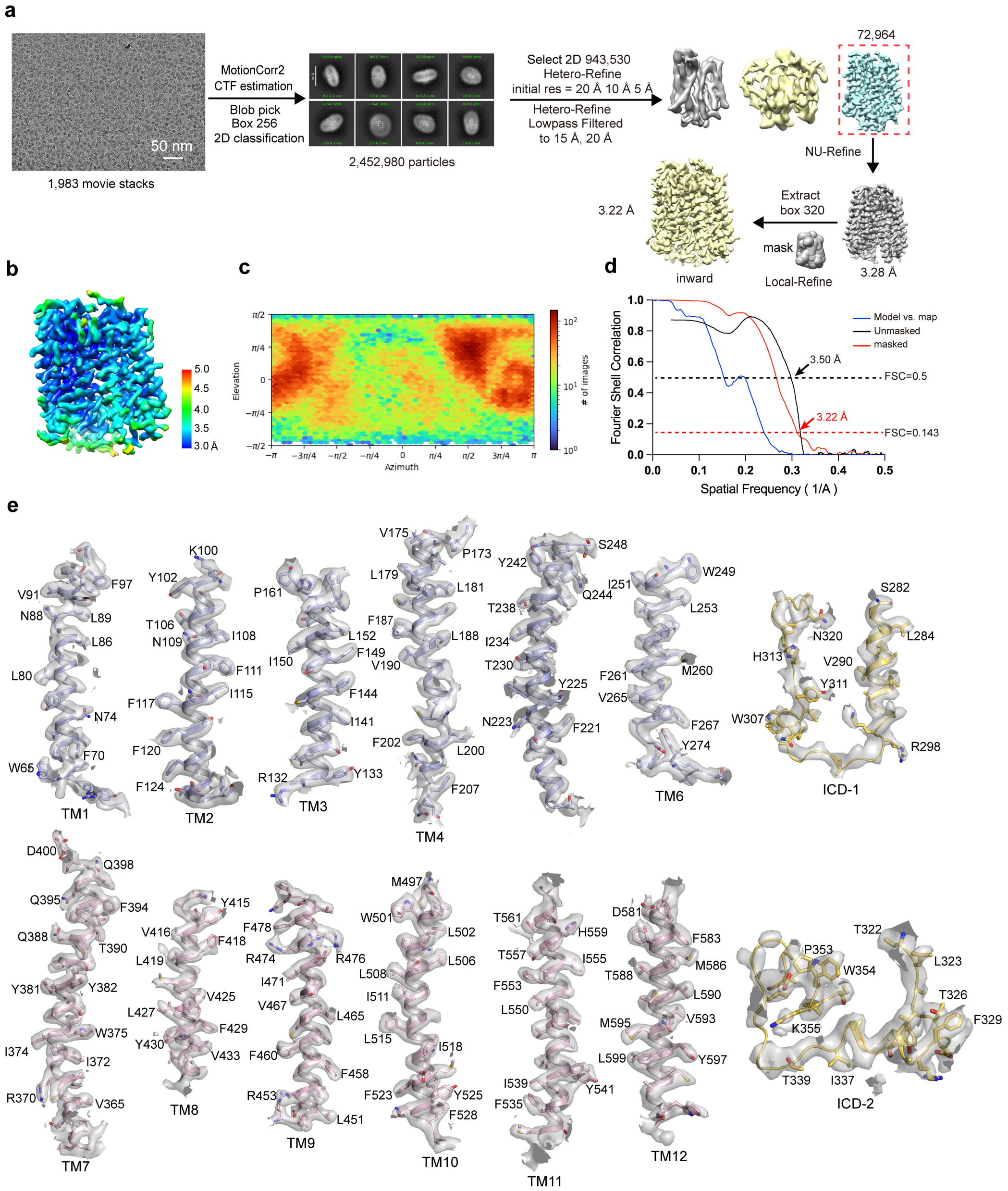

Supplementary Fig. S3. Cryo-EM analysis of GTR1^{inward}.

a, The flowchart for cryo-EM data processing of GTR1^{inward}. A representative motion-corrected EM micrograph (out of 1,983 micrographs) of GTR1^{inward}. Scale bar, 50 nm. **b,** Local resolution distribution of the sharpened maps of GTR1^{inward}. **c,** Particle angular distribution calculated in cryoSPARC for the final reconstruction of GTR1^{inward}. **d,** FSC curve of the final maps. **e,** Representative EM densities of GTR1^{inward}. EM densities are show in half-transparent surface. Side chains are shown as sticks and labelled. The map contour levels are at 5.5-8.5 σ with a carving range of 2.2-2.5 Å, respectively.

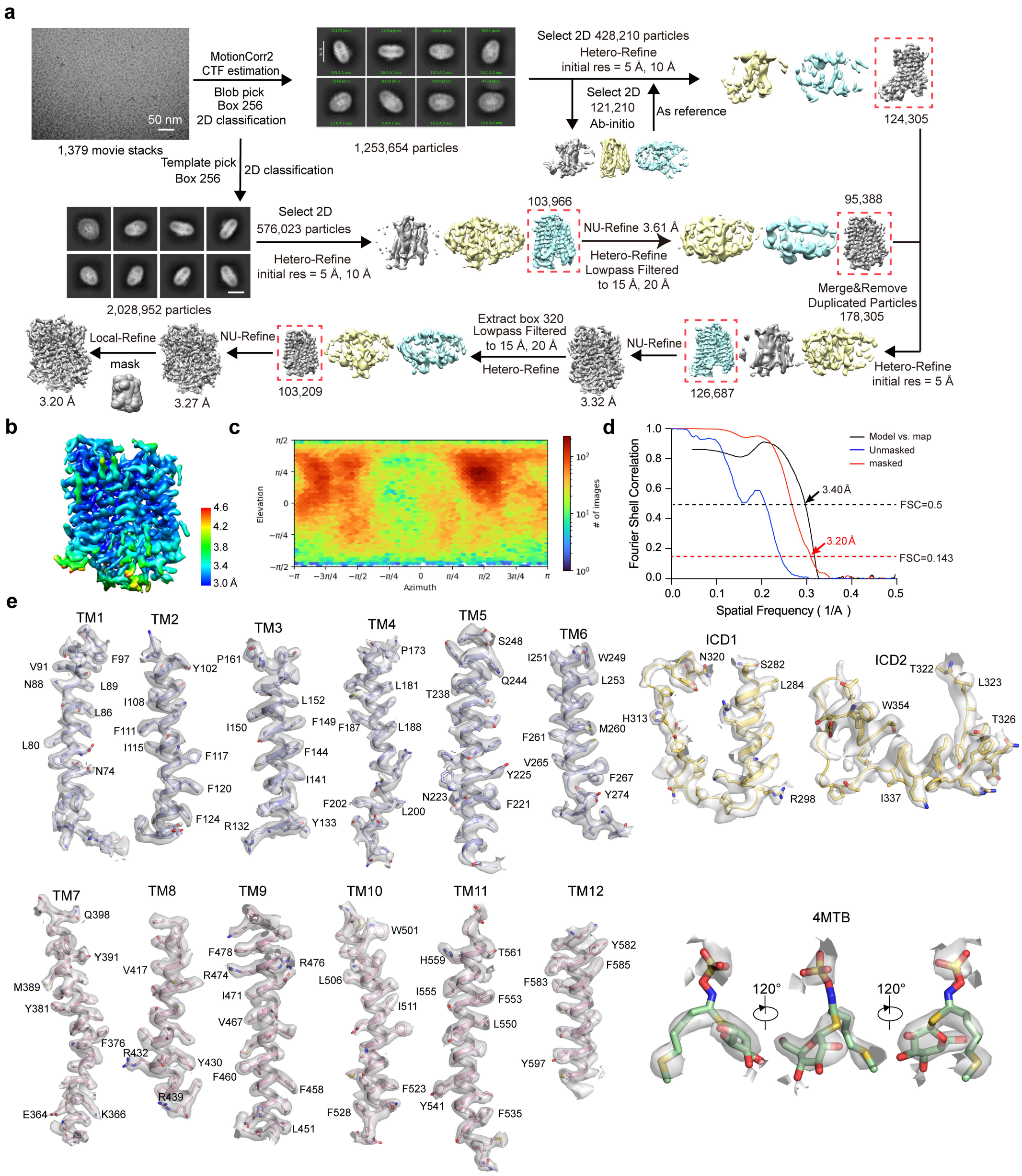

Supplementary Fig. S4. Cryo-EM data processing of GTR1^{4MTB}.

a, The flowchart for cryo-EM data processing of GTR1$^{4MTB.}$ A representative motion-corrected EM micrograph (out of 1,379 micrographs) of GTR1^{4MTB}. Scale bar, 50 nm. Scale bar in reference-free 2D class averages is 10 nm. **b,** Local resolution distribution of the sharpened maps of GTR1^{4MTB}. **c,** Particle angular distribution calculated in cryoSPARC for the final reconstruction of GTR1^{4MTB}. **d,** FSC curve of the final maps. **e,** Representative EM densities of GTR1^{4MTB}. Side chains are shown as sticks and labelled. EM densities are show in half-transparent surface. The map contour levels are at 4-7.5 σ with a carving range of 2.2 Å, respectively.

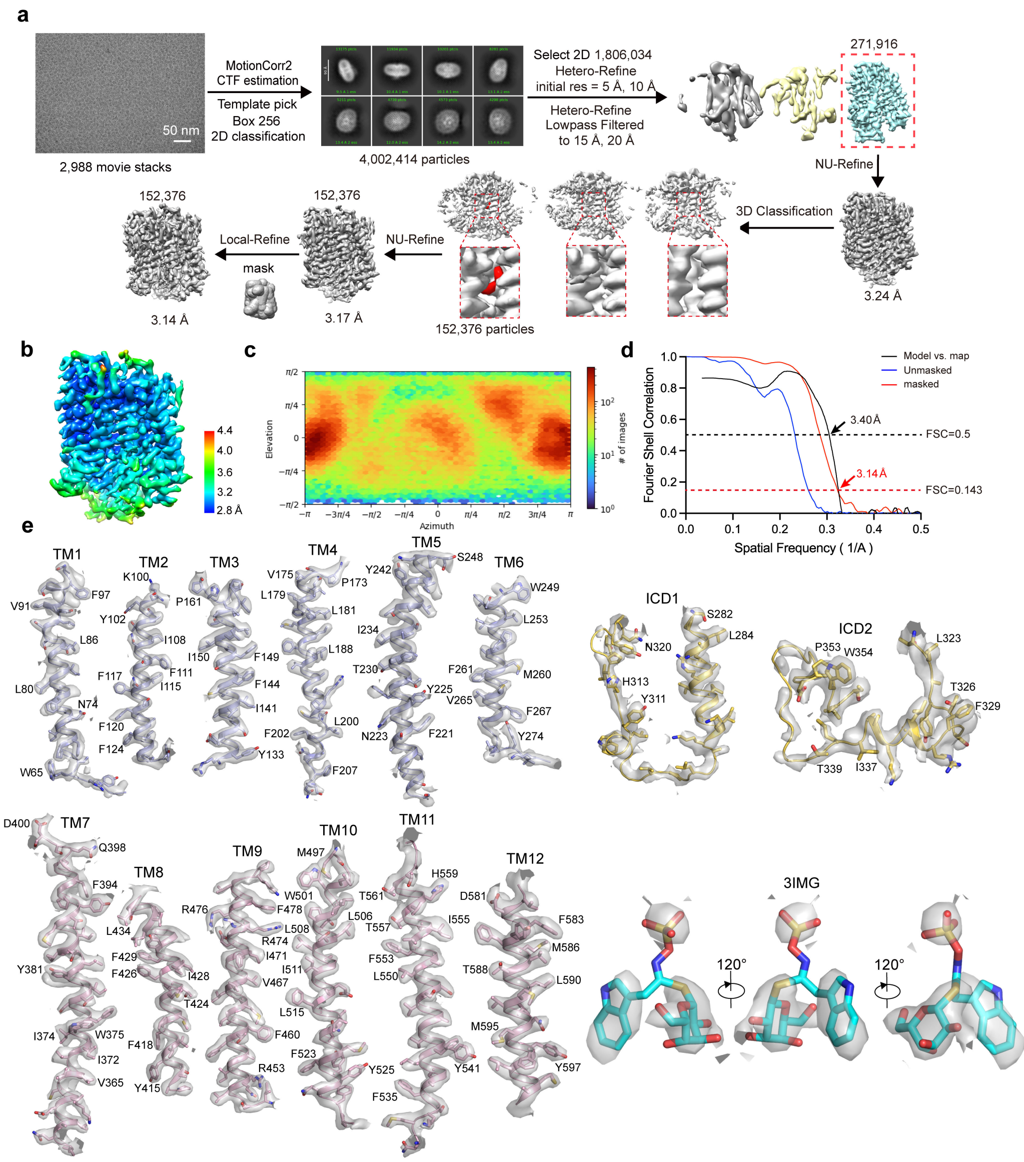

Supplementary Fig. S5. Cryo-EM data processing of GTR1^{3IMG}.

a, The flowchart for cryo-EM data processing of GTR1$^{3IMG.}$ A representative motion-corrected EM micrograph (out of 2,988 micrographs) of GTR1^{3IMG}. Scale bar, 50 nm. **b,** Local resolution distribution of the sharpened maps of GTR1^{3IMG}. **c,** Particle angular distribution calculated in cryoSPARC for the final reconstruction of GTR1^{3IMG}. **d,** FSC curve of the final maps. **e,** Representative EM densities of GTR1^{3IMG}. Side chains are shown as sticks and labelled. EM densities are show in half-transparent surface. The map contour levels are at 3.5-7.5 σ with a carving range of 2.2-2.5 Å, respectively.

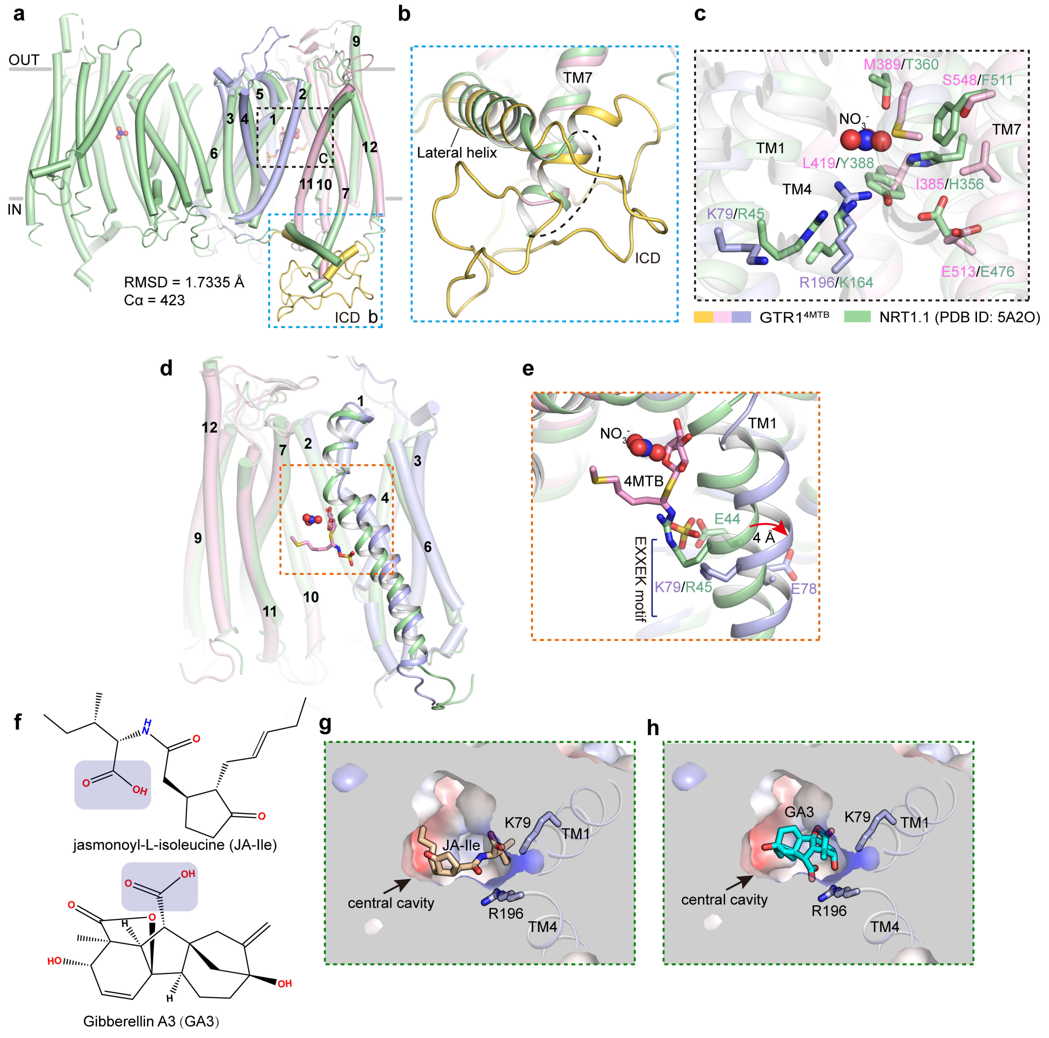

Supplementary Fig. S6. Structural comparisons of GTR1 and NRT1.1.

a, Superposition of GTR1^{4MTB} and the X-ray structure of NRT1.1 (green). Black and blue dashed lines box highlight the regions shown in panel b and c. **b** and **c**, Comparisons of the intracellular domain (**b**) and nitrate binding site (**c**). **d,** The different view from superpositions of GTR1 with monomeric NRT1.1. The orange dashed lines marking key areas in panel (e). **e,** Conformational shift in TM1 between GTR1^{4MTB} and NRT1.1. Red arrow indicates conformational shift of TM1. **f**, Chemical structures of jasmonoyl-L-isoleucine (JA-Ile) and gibberellin A3 (GA3). Dark squares indicate the negative moiety of the hormones. **g,h**, Fitting of JA-Ile (**g**) and GA3 (**h**) into the central cavity of GTR1. A cut-open electrostatic surface potential of GTR1 is shown (blue, positive; red, negative). JA-Ile and GA3 in brown and cyan sticks, respectively.

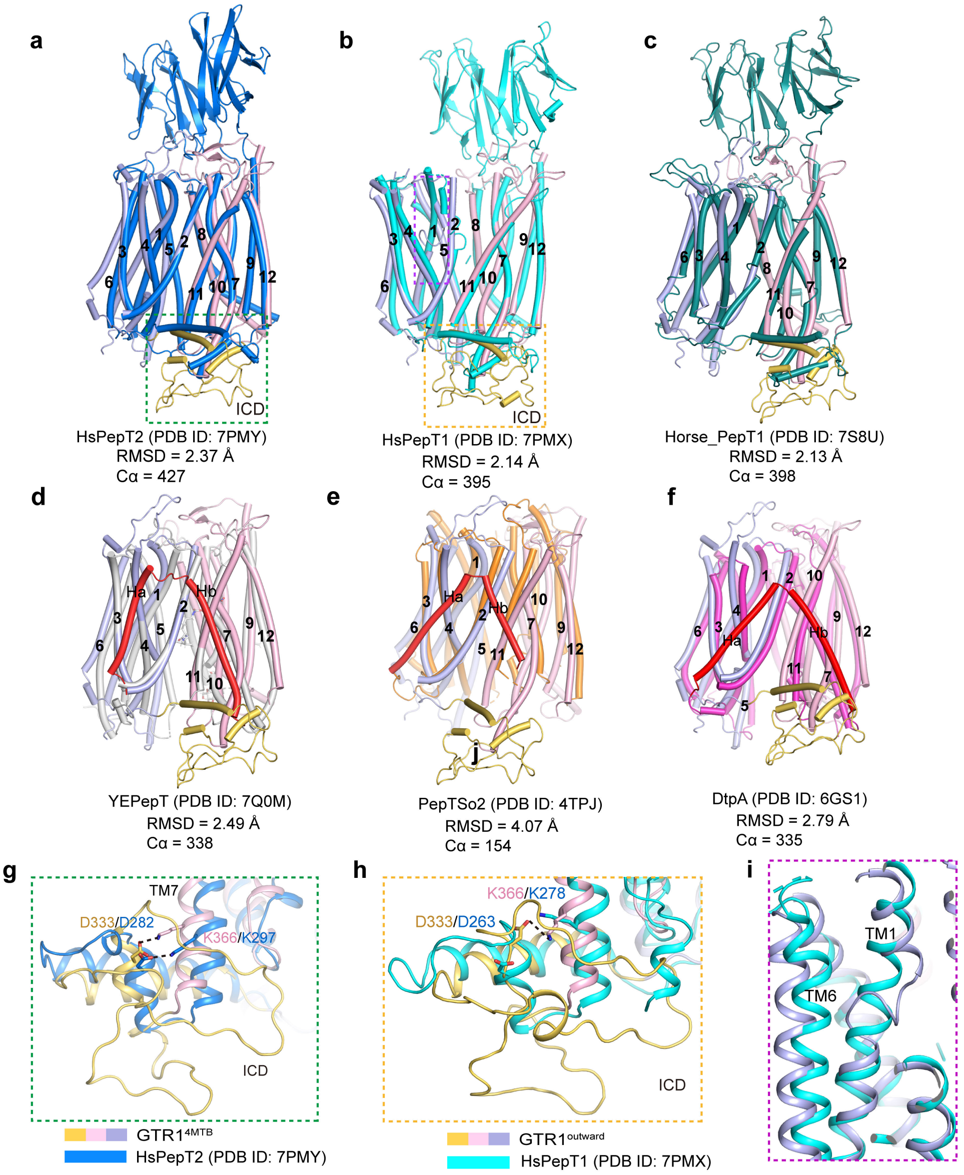

Supplementary Fig. S7: Structural comparisons of GTR1 with its homologs.

a-f, Superpositions of GTR1 with the structures of HsPepT2 (**a**), HsPepT1 (**b**), horse PepT1 (**c**), bacterial *Yersinia enterocolitica* YEPepT (**d**), *Shewanella oneidensis MR-1* PepTSo2 (**e**), and *Escherichia coli K-12* DtpA (**f**). Green dashed line box highlights the regions shown in panel **g**. **g** and **f,** Structural Comparisons of the intracellular domain (ICD) of GTR1 with that of HsPepT2 (**g**) and HsPepT1 (**h**). **i,** Structural comparisons of the TM1 of GTR1 with that of HsPepT1. GTR1 in purple and HsPepT1 in cyan.

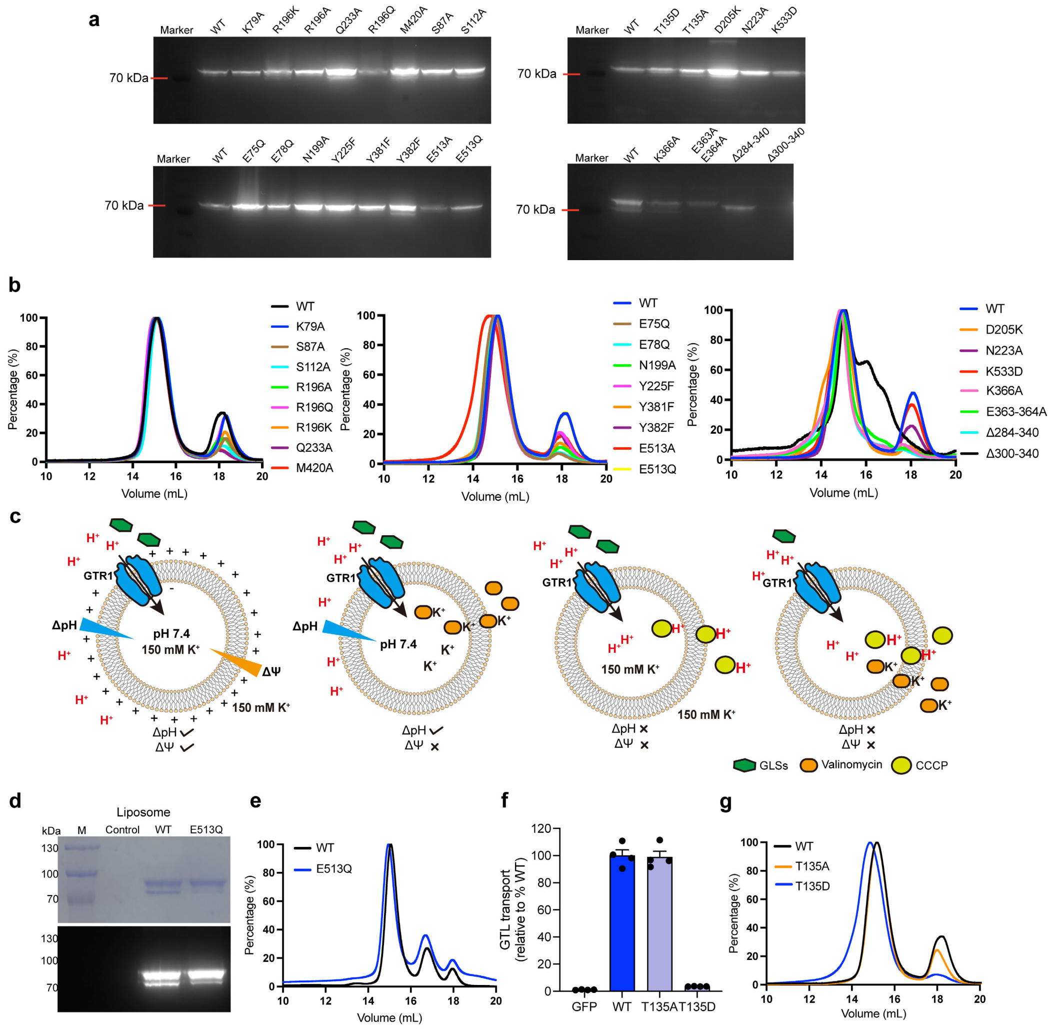

Supplementary Fig. S8. Biochemical profiles and proteoliposome assay of GTR1

a, In-gel fluorescence of GTR1^{WT} and its mutants. **b,** Fluorescence-detection size-exclusion chromatography (fSEC) profiles of GTR1^{WT} and mutants. **c,** A schematic of the experimental setup for liposome transport assay. Blue and orange triangles denote the condition with a proton gradient (ΔpH) and membrane potential (ΔΨ), respectively. The ΔΨ is dissipated, while the ΔpH is maintained (the second panel from the left). The two panels on the right represent conditions where the ΔpH is abolished, leading to equilibrated H^+ and K^+ concentrations across the membrane. This is achieved by in the presence of both CCCP alone or CCCP and valinomycin (which dissipates the H^+ gradient, resulting in a balanced state where no net K^+ gradient is established or required). Green hexagons represent the GLSs. Orange and yellow circles represent valinomycin and CCCP, respectively. **d**, Coomassie blue staining (top) and In-gel fluorescence (middle) of liposomes containing GTR1^{WT} or E513Q mutant. **e**, fSEC profiles of liposomes reconstituted GTR1^{WT} or E513Q mutant. **f,** GTL uptake by WT GTR1 and T135 mutants. GFP expressing cells serve as controls. Data were normalized to the total cell number for each variant and are presented relative to GTR1^{WT}. Values are mean ± s.e.m. of n = 4 independent replicates. **g,** fSEC profiles of GTR1^{WT} and mutants in panel f. A Superose 6 10/300 column was used for fSEC analysis.

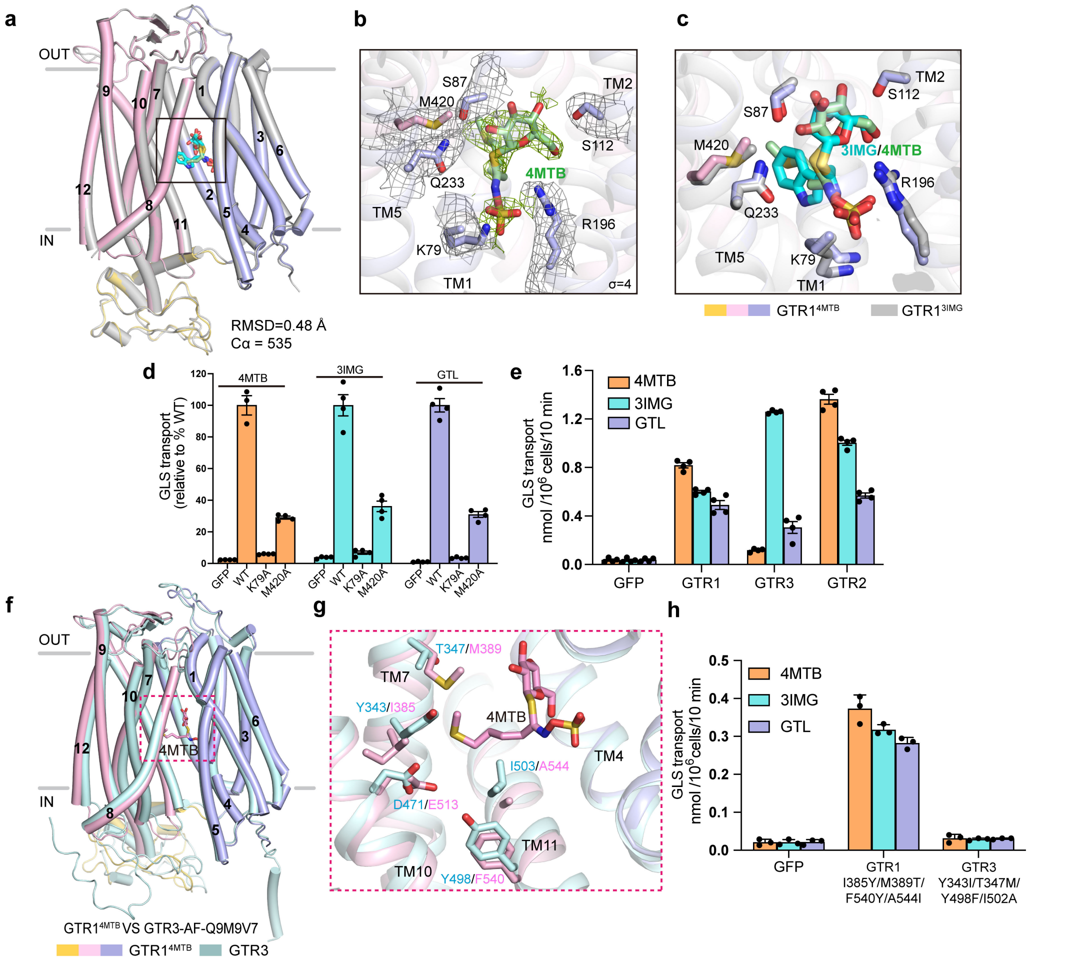

Supplementary Fig. S9. Structural basic of 4MTB recognition by GTR1

a, Superpositions of GTR1^{4MTB} with GTR1^{3IMG}. **b**, Interactions between 4MTB and GTR1. The density of residues and 4MTB are contoured at 4 σ shown in gray and green meshes. **c**, The superposition of the substrate-binding sites in GTR1^{4MTB} and GTR1^{3IMG}. **d**, Different substrates transport by WT and mutants of GTR1. **e,** GTL transport by GTR1, GTR2 and GTR3. **f,** Superpositions of GTR1 with GTR3. Pink dashed lines box highlights the region shown in panel g. **g,** Close-up view of the substrate-binding sites in GTR1 and GTR3. The non-conserved residues are depicted as sticks. **h,** GTL transport by mutants of GTR1 and GTR3. GFP served as controls. All data were normalized to the total cell number for each variant and are presented relative to GTR1^{WT}. Values are mean ± s.e.m. of n = 4 independent replicates.

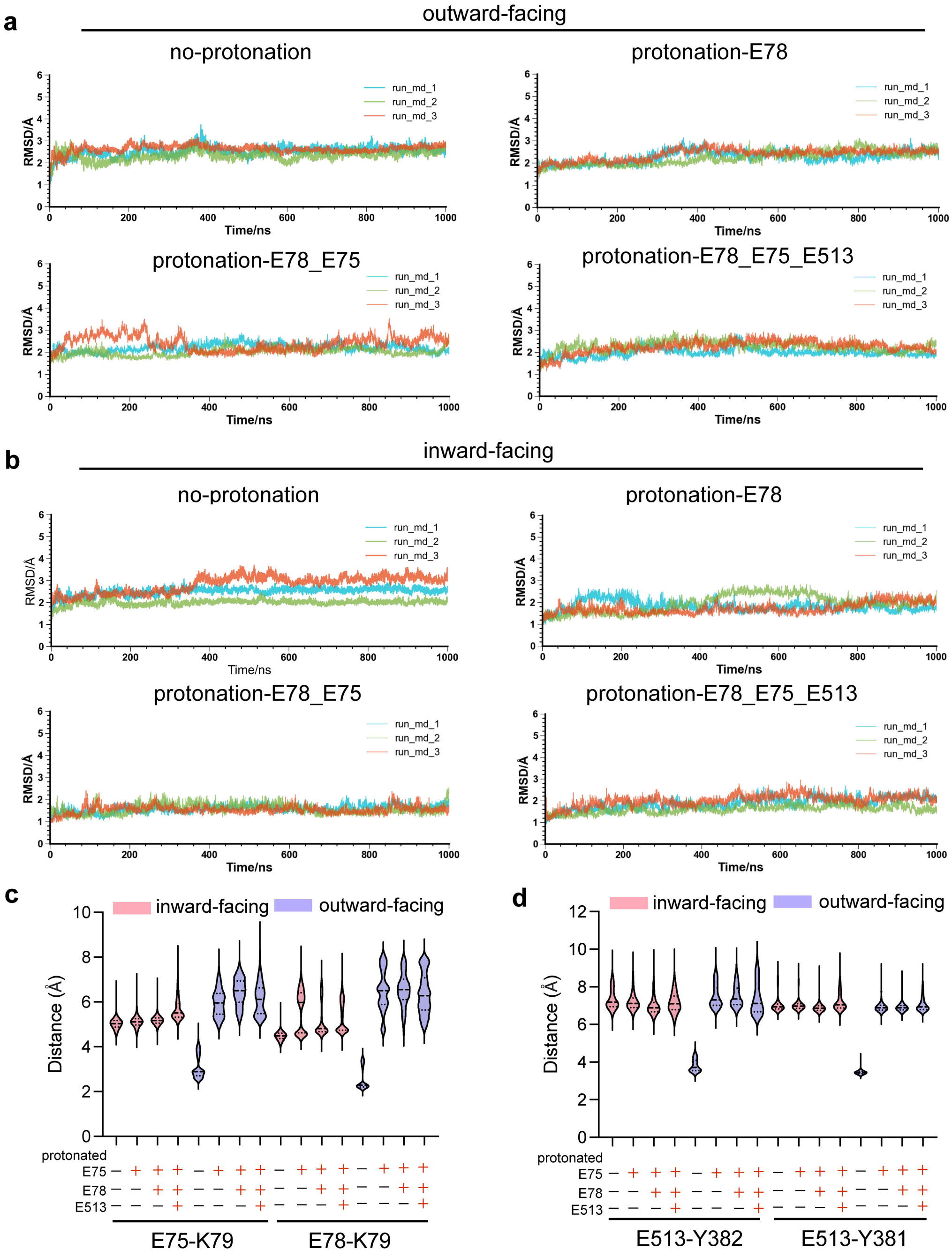

Supplementary Fig. S10 MD simulation analysis of proton-coupled dynamics in GTR1

a,b, Structural stability of the inward- (**a**) and outward-facing models (**b**) under protonated and non-protonated conditions in a membrane-like lipid bilayer environment. Each simulation was performed for a duration of 1 μs with three independent repeats. **c,d,** Distance analysis of key residue pairs during MD simulations. Distances for Glu75-Lys79 and Glu78-Lys79 (**c**), Glu513-Tyr382 and Glu513-Tyr381 (**d**) were measured under both protonated and non-protonated conditions for the inward-facing (pink) and outward-facing (purple) states. The black plus sign indicates the non-protonated state, and the red minus sign indicates the protonated state. Distance values are mean ± s.e.m. of n = 3 independent replicates.

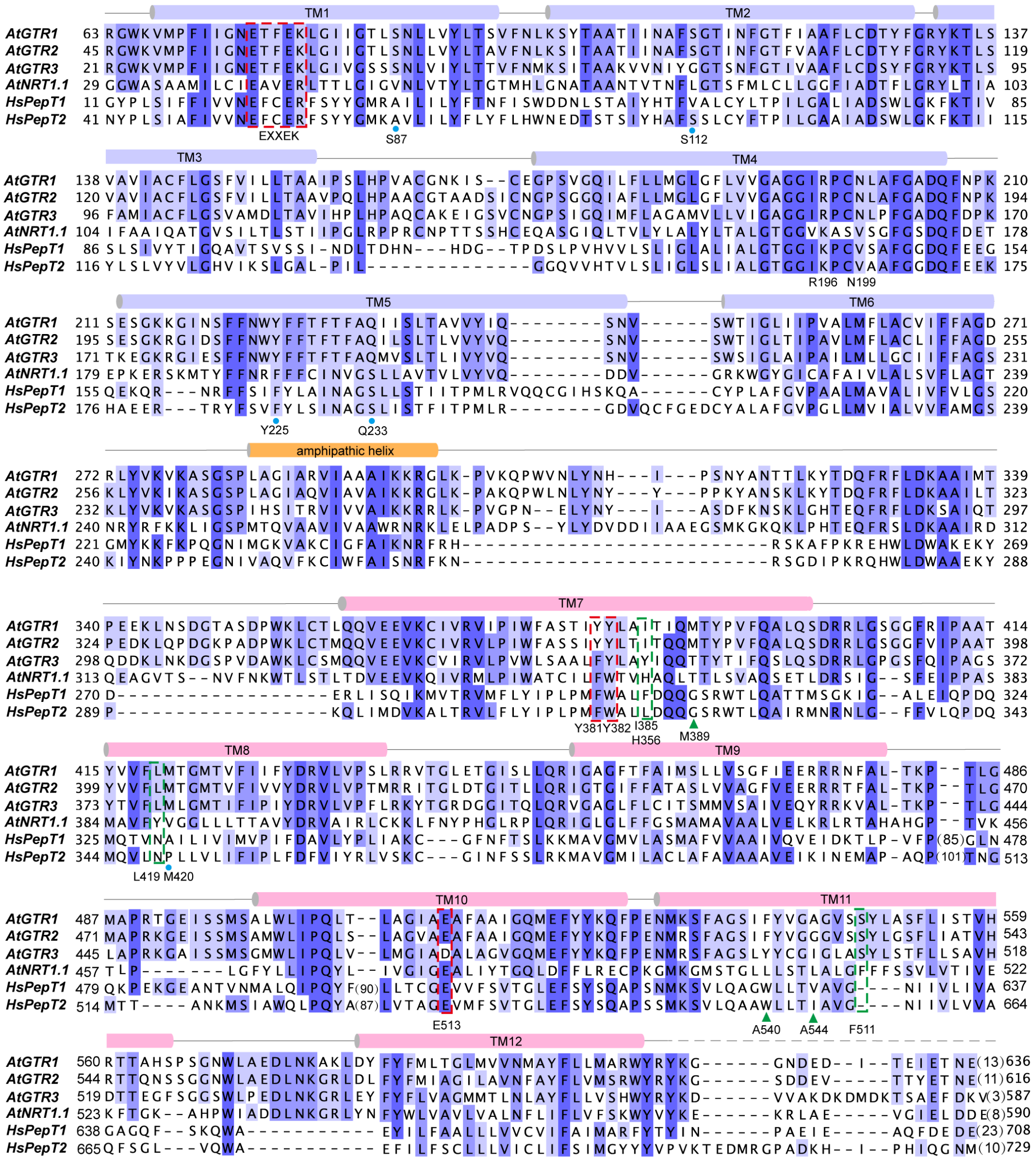

Supplementary Fig. S11. Sequence alignment of GTR1 homologs.

Sequence alignments of GTRs and NRT1.1 from *Arabidopsis thaliana*, PepT1 and PepT2 from human. Secondary structural elements are labelled on the top of the sequences. The red dashed and blue circle highlight residues involved in proton-coupling and substrate-binding sites. The green triangle marks non-conserved residues between GTR1 and GTR3 about substrate-binding sites. The green dashed highlights residues indicated in NRT1.1.

Supplementary Table S1. Cryo-EM data collection, refinement, and validation statistics

	GTR1-4MTB (EMDB-64193) (PDB: 9UIF)	GTR1-3IMG (EMDB-64204) (PDB: 9UIT)	GTR1-inward (EMDB-64185) (PDB: 9UI6)	GTR1-outward (EMDB-64181) (PDB: 9UI1)
**Data collection and processing**				
Magnification			105,000 ×	
Voltage (kV)			300	
Electron exposure (e^{-}/Å^2)			60	
Defocus range (μm)			−1.0 ~ −2.0	
Pixel size (Å)			0.85	
Symmetry imposed			C1	
Initial particle images (no.)	1,253,654	4,002,414	2,452,980	1,104,702
Final particle images (no.)	103,209	152,376	72,964	54,982
Map resolution (Å)	3.20	3.14	3.22	3.54
FSC threshold	0.143	0.143	0.143	0.143
Map resolution range (Å)	3.0 ~ 4.6	3.14 ~ 3.21	3.5 ~ 5.5	3.0 ~ 5.0
**Refinement**				
Initial model			Alphafold2	
Model resolution (Å)	3.40	3.40	3.50	3.80
FSC threshold	0.5	0.5	0.5	0.5
Map sharpening *B* factor (Å^2)	-116.6	-138.5	-109.5	-123.7
Model composition				
Non-hydrogen atoms	4,259	4,269	4,222	4,085
Protein residues	547	548	547	539
Ligands	21	19	14	7
B factors (Å^2)				
Protein	59.03	66.53	75.28	79.65
Ligand	86.42	98.07		
R.m.s. deviations				
Bond lengths (Å)	0.003	0.003	0.003	0.003
Bond angles (°)	0.569	0.548	0.574	0.622
Validation				
MolProbity score	1.49	1.41	1.43	1.64
Clashscore	4.69	4.21	8	6.40
Poor rotamers (%)	0	0	0.23	0.24
Ramachandran plot				
Favored (%)	96.33	96.70	96.33	95.89
Allowed (%)	3.49	3.11	3.67	4.11
Disallowed (%)	0.18	0.18	0	0
